# Oral delivery of insulin with intelligent glucose-responsive switch for blood glucose regulation

**DOI:** 10.1186/s12951-020-00652-z

**Published:** 2020-07-14

**Authors:** Xia Zhou, Hongwei Wu, Ruimin Long, Shibin Wang, Haiwang Huang, Yanhua Xia, Pei Wang, Yifeng Lei, Yuanyuan Cai, Duanhua Cai, Yuangang Liu

**Affiliations:** 1 College of Chemical Engineering, Huaqiao University, Xiamen, 361021 China; 2 Department of Chemistry, Xinxiang Medical University, Xinxiang, 453003 Henan China; 3 Institute of Pharmaceutical Engineering, Huaqiao University, Xiamen, 361021 China; 4 Fujian Provincial Key Laboratory of Biochemical Technology, Huaqiao University, Xiamen, 361021 China; 5 Internal Medicine Department, Xiamen Haicang Hospital, Xiamen, 361000 Fujian China; 6 The Institute of Technological Sciences & School of Power and Mechanical Engineering, Wuhan University, Wuhan, 430072 China

**Keywords:** Glucose-responsive, Hypoxia-sensitive, Self-assembly nanoparticles, Oral insulin delivery

## Abstract

**Background:**

The traditional treatment for diabetes usually requires frequent insulin injections to maintain normoglycemia, which is painful and difficult to achieve blood glucose control.

**Results:**

To solve these problems, a non-invasive and painless oral delivery nanoparticle system with bioadhesive ability was developed by amphipathic 2-nitroimidazole–l-cysteine–alginate (NI–CYS–ALG) conjugates. Moreover, in order to enhance blood glucose regulation, an intelligent glucose-responsive switch in this nanoparticle system was achieved by loading with insulin and glucose oxidase (GOx) which could supply a stimulus-sensitive turnover strategy. In vitro tests illustrated that the insulin release behavior was switched “*ON*” in response to hyperglycemic state by GOx catalysis and “*OFF*” by normal glucose levels. Moreover, in vivo tests on type I diabetic rats, this system displayed a significant hypoglycemic effect, avoiding hyperglycemia and maintaining a normal range for up to 14 h after oral administration.

**Conclusion:**

The stimulus-sensitive turnover strategy with bioadhesive oral delivery mode indicates a potential for the development of synthetic GR-NPs for diabetes therapy, which may provide a rational design of proteins, low molecular drugs, as well as nucleic acids, for intelligent releasing via the oral route.
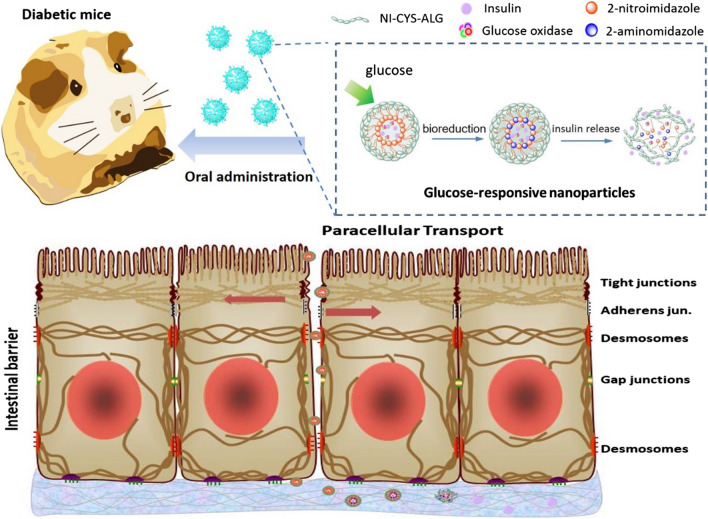

## Introduction

More than 425 million people suffered from diabetes mellitus (DM) in the world in 2017 according to a report from the 8th edition of the International Diabetes Federation (IDF) Diabetes Atlas, making the management of diabetes one of the largest worldwide health challenges [1]. Diabetes, especially type 1 diabetes characterized by a high blood glucose levels, can result in several complications and may even be life-threatening. The goal of management of type 1 diabetes is persistent glycemic control for patients within healthy euglycemic ranges of blood glucose levels (70–140 mg/dL or 4–8 mM) [2]. Recently, the report of a chemically modified alginate derivative which could protect viable and glucose-responsive allogeneic islets for 4 months without the need for immunosuppression, brings a promising to enable the long-term transplantation of islets for the correction of insulin deficiency [3]. However, owing to uncontrollable vigorous foreign-body responsive, rare matching type and expensive cost, the long-term glycemic control of diabetes is still widely used by insulin not transplantation of islets. Moreover, methods of administration including “closed-loop” insulin delivery system [4], insulin-loaded matrix device [5] and so on are insulin delivery systems able to “secrete” insulin in response to blood glucose levels that can improve quality of life. Although significant developments have been made in insulin therapy over the past few decades through traditional care, i.e. subcutaneous injection for people with diabetes, which often is lacking adequate control of blood glucose and includes painful injections to maintain normoglycemia [6], there is need to explore a non-invasive insulin delivery system for diabetes therapy. Various strategies have emerged to be studied, such as a nasal management route, lung drug delivery, micro-array patch and so on. Those strategies are currently lacking due to low drug penetration, cilia scavenging effect, indeterminate biological safety and inconvenient management, which means they are not optimal for drug delivery of insulin. For the safety and adequate utilization of concise, oral drug delivery, it will need to be easy for compliance and have an easy to use route for long-term drug delivery [7, 8].

Nevertheless, there are lots of major hurdles that must be overcome before peptides and bioactive proteins can be successfully delivered via the oral route, such as product stability, gastrointestinal (GI) stability and absorption [9]. In order to improve the effective absorption of oral protein and peptide drugs, strategies including structure modification of proteins and addition of absorbent promoter and enzyme inhibitors are widely used. However, challenges related to such means still persist, the biological activity of a protein may be reduced and/or the pharmacokinetics may be changed after structural modification [10]. In addition, irreversible damage to the gastrointestinal tract can be caused by using an absorption promoter for long term. Besides, the resulted gastrointestinal disorder may be associated with an enzyme inhibitor. Lately, the report of an ionic liquid-based oral formulation of insulin achieved an unprecedented improvement in oral bioavailability of insulin and brings a promising forward to achieve the insulin oral route of administration [11]. More important, except painlessness, adjusting insulin release according to the concentration of blood glucose is so important that can decrease the multiple side effects from diabetes. These shortcomings prevent these strategies from being ideal strategies to effectively promote oral absorption of protein and peptide drugs. According to recent research, the major considerations for oral insulin administration are: (i) keeping the stability and activity of insulin in GI tract; (ii) improving the adhesion ability of drug carrier to prolong the resistant time in the intestine, which can promote drug permeation; and (iii) adjusting drug releasing behavior according to the concentration changes of blood glucose, which can achieve intelligent glucose management, thus, decreasing the multiple side effects from diabetes [12–14]. Nanoparticles can solve these problems, especially with addition of an adhesive substance, i.e. thiol groups. Sulfhydryl modified nanoparticles cannot only encapsulate bioactive proteins so as to protect them during storage and after ingestion, but have the ability to prevent enzyme degradation as well as promote adhesion of the carrier to improve drug paracellular uptake overcoming epithelial cell barrier [15]. At present, bioadhesive nanoparticles are considered an innovative method for oral drug delivery.

Importantly, how to execute insulin release in blood intelligently needs to be determined. Focusing on the development of controlled insulin release, glucose-responsive insulin delivery is beneficial to reduce the drawbacks associated with traditional methods, which can achieve “smart” insulin release according to blood glucose concentration via a glucose-responsive insulin release system adjusted by related functional sections such as phenylboronic acid (PBA) [16], glucose binding proteins (i.e. Con A) [17] and GOx [18]. Herein, we proposed a non-invasive and painless oral delivery system developed by designing an intelligent glucose-responsive switch for blood glucose regulation, according to an in situ stimulus-sensitive turnover strategy. Thus, we designed a 2-nitroimidazole–l-cysteine–alginate (NI–CYS–ALG) polymer with functional elements as follows: 2-nitroimidazole (NI) [19], which is the most popular nitroimidazole derivative for hypoxia targeting imaging, is expected to be bio-reduced from a hydrophobic component to hydrophilic 2-aminoimidazoles under the local hypoxic conditions caused by GOx in the hyperglycemia, providing hypoxia-sensitive ability [20]; l-cysteine [21], including thiol-groups, provides improved pH stability and prolonged resistance time in the mucus layer of the GI tract, which is believed to interact with cysteine-rich subdomains of mucus glycoproteins and has, therefore, proven to be a potential “pharmaceutical glue” [22]. In addition, cysteine can also improve intestinal permeability via paracellular route by changing the distribution of cytoskeletal F-actin and ZO-1 protein. Hence, the resulting GR-NPs based on the formation of a thiolated polymer conjugated with nitroimidazole derivatives displaying a prolonged residence time on gastrointestinal mucosal epithelial without enzyme degradation can be further used for oral administration. Hence, we expected this stimulus-sensitive turnover tactics to be considered as a rational design of medically functional bioactive proteins for intelligent releasing via the oral route.

Herein, we highlight this “smart” glucose-responsive nanoparticles system as following:

i Undergo structural transformations adjusted by glucose concentration changes, leading to glucose-stimulated insulin release.
j Oral management enhanced by the addition of thiol groups could improve intestinal permeability and enzyme inhibition.
k Combined with ease of administration, biocompatibility and “smart” responsiveness without hypoglycemia.

## Experiment section

### Materials

Sodium alginate (ALG), 2-nitroimidazole (NI) and 6-(Boc-amino)hexyl bromide were purchased from Sigma-Aldrich Chemical Company (St. Louis, MO, the USA). l-cysteine (CYS), 1-(3-dimethylaminopropyl)-3-ethylcarbodiimide hydrochloride (EDC) and *N*-hydroxysuccinimide (NHS), were all purchased from Aladdin Bio-Chem Technology Co., Ltd (Shanghai, China). Glucose oxidase (GOx), insulin (INS) and fluorescein isothiocyanate labeled insulin (FITC-INS), used as model drugs, were purchased from Dalian Meilun Biotechnology Co., Ltd (China). The model cells (Caco-2 and HT-29) were got from American type culture collection (ATTC) (Shanghai, China), Male ICR mice and SD rats were supplied by Wushi Laboratory Animal Co., Ltd (Fuzhou, China).

### Synthesis of NI–CYS–ALG polymers

The NI derivative (NI-NH_2_, details in Additional file 1) was conjugated to l-cysteine–alginate sodium (CYS–ALG) in the presence of 1-(3-dimethylaminopropyl)-3-ethylcarbodiimide hydrochloride (EDC) and *N*-hydroxysuccinimide (NHS). In brief, cysteine-alginate (0.1 g) was dissolved in a 5 mL mixture of formamide and dimethyl formamide (DMF) (1:1, *v/v*), after which 50 mM EDC and NHS were added and stirred for 15 min. The NI derivative (0.1 g) in 1 mL DMF was slowly added to the reaction mixture and stirred for 1 day. The resulting solution was dialyzed against an excess of water/methanol (1/1–1/3, *v/v*) for 1 day and against distilled water for 2 days at room temperature before being lyophilized. The molecular weight of dialysis membrane was 3500. Samples were lyophilized and then stored at 4 °C until further use. The amount of the NI was detected at 330 nm by a UV–vis spectrophotometer (details in Additional file 1).

### Characterization of polymers by ^1^H NMR and FTIR

The chemical conjugation of NI derivate, CYS–ALG and 2-nitroimidazole–l-cysteine–alginate sodium (NI–CYS–ALG) were confirmed using 500 MHz nuclear magnetic resonance (NMR) spectroscopy and FTIR spectroscopy. Samples for analysis were prepared in deuterated chloroform (CDCl_3_), deuterated methanol (MeOD) and deuterium oxide (D_2_O) and detected by ^1^H NMR spectroscopy (Bruker AVANCE III 500, Germany). Fourier transform infrared (FTIR) spectra were recorded on a FTIR spectrometer (Thermo Scientific Nicolet iS 50, America) using the KBr pellets method in the wavenumber range of 400–4000 cm^−1^.

### Preparation of GR-NPs and insulin-loaded GR-NPs

First, the terminal NI–CYS–ALG (20 mg) was dissolved in 20 mL methanol/water (1/1–1/2, *v/v*), and the reaction proceeded under stirring for 30 min at room temperature. Second, the mixture was self-assembled nanoparticles under ultrasonic cell breakup instrument (parameter: 100–200 w, 5 s/2 s) for 5 min at 0 °C. Later, the suspension was centrifuged at 10,000 rpm for 30 min under 4 °C and the precipitate was re-suspended in deionized water (10 mL). The insulin (INS)/glucose oxidase (GOx)-loaded glucose-responsive nanoparticles (GR-NPs) were prepared by similar route except the addition of a certain amount of insulin (10 mg) and GOx (2 mg). The content of insulin was detected by HPLC method. The parameters of HPLC (Shimadzu LC-20A, Japan) were containing a C18 column, 5 μm, 4.6 × 150 mm (Diamond ODS, Korea) and a UV detector set at λ_max_ of 214 nm. The mobile phase comprised 0.1 M NaH_2_PO_4_ buffer (pH 3.0) and acetonitrile (containing 0.1 M Na_2_SO_4_) (72:28, *v/v*), degassed and then ran at a flow rate of 1.0 mL/min at 27 °C. The injection volume was 20 μL (details in Additional file 1). The drug loading capacity (DLC) and encapsulation efficiency (EE) shown in Additional file 1: Figure S6 were calculated by the following equations. The different weight ratios of CYS/ALG (1:2, 1:1, 2:1) were contributed to GR-NPs 1, GR-NPs 2 and GR-NPs 3, respectively.

1 $$\begin{array}{*{20}c} {\text{Drug}\; \text{loading} \;\text{capacity} \left( {{\text{DLC}}, \% } \right) = \frac{{W_{entrap} }}{{W_{particles} }} \times 100} \\ \end{array}$$

2 $$\begin{array}{*{20}c} {\text{Encapsulation}\; \text{efficiency} \left( {{\text{EE}}, \% } \right) = \frac{Actual\; drug\; content}{Theoretical\; drug\; content} \times 100} \\ \end{array}$$

where W_entrap_ = weight of drug in GR-NPs and W_particles_ = weight of GR-NPs.

### Observation of morphology and detection of size distribution and zeta potential

The proper amount of GR-NPs (the weight ratio of CYS/ALG, 2:1) solution was diluted to a certain concentration, then dropped onto the copper net coated with carbon film (support film), where it stayed for 2 to 3 min and was then dripped with 1% phosphotungstic acid solution after being wiped with filter paper. GR-NPs were then observed by transmission electron microscope (Hitachi H-7650 TEM, Japan). The freeze dried GR-NPs were resuspended in PBS solution. The particle size and zeta potential of GR-NPs were measured by laser particle size and zeta potential analyzer (Malvern ZetaPALS, Britain) (Additional file 1: Table S2). The dispersion stability of GR-NPs (CYS:ALG, 2:1) was measured during different times (1 day, 3 days, 5 days).

### In vitro enzymatic inhibition

Pepsin and trypsin were used as model proteases to investigate the enzymatic inhibition of GR-NPs in vitro. Pepsin and trypsin were added into different buffers with pH 1.2 and 6.8 to obtain simulated gastric fluid (SGF, containing pepsin) and simulated intestinal fluid (SIF, containing trypsin) solutions, respectively. GR-NPs were added into SGF and SIF solutions, incubated at 37 °C at 100 rpm for a certain time. Supernatant samples were collected at a predetermined time after centrifugation for 10 min at 12,000 rpm and detected by HPLC.

### Glucose responsive capability of GR-NPs

To examine the glucose stimuli-responsive ability, GR-NPs were incubated in different PBS buffers containing different concentration of glucose (0, 100, 400 mg/dL), the pH values were measured by pH meter, the size of GR-NPs were confirmed by dynamic light scattering (DLS) measurement and the contents of NI were detected by UV–vis spectrophotometer within 6 h. The morphology changes of GR-NPs were observed via TEM observation. Oxygen consumption was determined by oxygen consumption assay kit.

### Cumulative insulin release of GR-NPs in vitro

The in vitro drug release behavior of nanoparticles was studied by dialysis (MWCO 8000–14000). The drug release behavior in different pH PBS buffer solutions (pH 1.2, 4.9, 6.8, 8.0), different glucose concentrations (0, 100, 400 mg/dL) and alternately changing glucose concentration was investigated. The content of insulin was determined by HPLC, the cumulative release of insulin was measured and the release curve was drawn.

### Characterization by CD spectrum

The secondary structure of the insulin released from GR-NPs was further examined by a circular dichroism (CD) spectrometer J-810 (JASCO Corporation, Japan), which could evaluate the conformational stability of proteins. For comparison of released insulin, pristine insulin was used as control. Using PBS (pH 7.4) as solvent, insulin solution and insulin released from GR-NPs were prepared, with a final concentration of insulin of 80 μg/mL. By CD, the scanning range was 190–250 nm, the precision was 0.2 nm, the bandwidth was 1.0 nm and the scanning rate was 200 nm/min.

### Cell culture

Caco-2 cells were cultured in low glucose DMEM supplemented with 10% fetal bovine serum. First, the culture medium was removed, the residual serum was removed by PBS rinsing, 1.0 mL 0.25% trypsin-EDTA was added for digestion and the cell layer was separated under microscope. If no suspension was observed, then 10% serum was added to the medium to terminate the cell digestion. After centrifugation, 6.0–8.0 mL complete culture medium was added, and the cells were gently blown. A certain amount of cell suspension was added to a new cell culture flask and cultured at 37 °C with 5% CO_2_. Different contents of GR-NPs (0 to 1000 μg/mL) were incubated with Caco-2 cells in different times (24 h, 48 h and 72 h), the cell biocompatibility detection was determined by MTT assay. The cell penetrating properties of the GR-NPs were evaluated by confocal laser scanning microscope (CLSM) after different incubation times (30 min, 1 h or 2 h) with Caco-2 cells. Cells were counterstained with DAPI (blue) and insulin was labeled by FITC (green). Moreover, F-actin on the membrane of cells was stained with TRITC Phalloidin, cell adhesion detection was also measured by CLSM observation after incubating for 30 min with FITC-insulin loaded GR-NPs and insulin alone (control) (Additional file 1: Figure S9). In addition, the interaction of GR-NPs and membrane of Caco-2 cells was detected by the distribution of F-actin, ZO-1 and occluding protein, after being cultured with insulin and GR-NPs for 2 h, the tight junction protein ZO-1 and occludin on Caco-2 cell signed by FITC was observed by CLSM. The group of INS was the control.

### Animal experiments

Healthy male SD rats were randomly divided into six groups (five rats per group) after fasting for 12 h before the experiment. Diabetic rats were induced by injection of streptozotocin (STZ, 70 mg/kg) dissolved in a 10 mM citrate buffer (pH 4.5) for 3 days. Rats with fasting blood glucose concentration of more than 16.6 mM (almost 22–30 mM) after an additional week were considered as a diabetic model and could be used for further investigations. Blood glucose level was determined using a glucose meter (Accu-Chek^®^ Performa, Roche, Germany). For the analysis of plasma insulin levels after administrated with samples (5 IU/kg insulin solution for subcutaneous injection on diabetic rats; 75 IU/kg insulin solution, INS-loaded NPs, INS/GOx GR-NPs and saline solution for oral administration on diabetic rates; INS/GOx loaded GR-NPs for oral administration on normal rats), blood samples were centrifuged at 3000 rpm for 15 min and, subsequently, quantified using an appropriate insulin ELISA kit. In order to investigate the permeation and the absorption of insulin at the position of small intestine, insulin solution (50 IU/kg) and INS/GOx GR-NPs (50 IU/kg insulin) were delivered by ileal administration in rats.

Blood glucose level and plasma insulin level were detected after 0 h, 1 h, 2 h, 3 h, 4 h administration. In order to observe the GR-NPs in vivo, diabetes rats were orally delivered by fluorescein isothiocyanate labeled insulin (FITC-INS)/GOx loaded GR-NPs (50 IU/kg), then were observed under living imaging systems (IVIS Lumina II, Caliper Life Science, the USA) with the following time at 1 h, 2 h, 3 h, 4 h. Similarly, small intestines of rats were observed by CLSM after 0.5 h, 1 h, 2 h, 4 h oral FITC-INS/GOx loaded GR-NPs administration. The permeability efficient value (Papp) of FITC-INS from GR-NPs was measured by the equation:

$$P_{app} = \left( {\frac{\Delta Q}{\Delta t}} \right) \times \left( {\frac{1}{{A \times C_{0} }}} \right)$$

P_app_, apparent permeability (cm/s); ΔQ/Δt, permeability rate (amount of FITC-INS traversing the cell layers over time); A, diffusion area of the layer (cm^2^); C_0_, apically added FITC-INS concentration.

FITC-INS/GOx loaded GR-NPs and FITC-INS were encapsulated to intestinal tissue (both ends were binding, 20 cm) and then were exposed to Krebs–Ringer for different times (1 h, 2 h, 3 h, 4 h). The content of FITC-INS was investigated by fluorescence spectrometer (F900/FLS920, EDINBURGH, China). After GR-NPs and saline for 3 h oral administration, rats/mice were sacrificed respectively. The intestinal tissue slides were observed by CLSM via H&E staining. The intestinal tissue of mice penetrating properties of the FITC-INS/GOx loaded GR-NPs were evaluated by confocal laser scanning microscope (CLSM) after different oral times (10 min, 30 min, 1 h or 2 h). Similarly, the small intestine adhesion abilities of rats were detected by CLSM after different oral times (0.5 h, 1 h, 2 h, 4 h) and the different sites of the small intestine (duodenum, ileum, jejunum and colon) were measured following 2 h oral administration. Tissues were counterstained with DAPI (blue) and insulin was labeled by FITC (green).

### Statistical data analysis

Statistical data analyses were performed using one-way ANOVA to determine significant differences among groups, after which the Student’s t-test was used for comparison between individual groups. *P < 0.05, **P < 0.01 were considered as the level of significance.

## Results and discussion

### Synthesis and characterization of GR-NPs

The GR-NPs were formed by self-assembly with the NI–CYS–ALG polymer, encapsulating insulin and GOx in the core. The NI–CYS–ALG polymer was formed by l-cysteine, amine-functionalized nitroimidazole and alginate sodium by an amidation reaction via EDC/NHS (details in Additional file 1: Figure S1). Briefly, l-cysteine was first conjugated to the backbone of protonated alginate to obtain CYS–ALG conjugate (with different CYS/ALG weight ratios), including multiple thiol groups that can enhance bioadhesive ability of the GR-NPs, which have a capability to prolong the resistant time of drug in the epithelia. Secondly, CYS–ALG was covalently modified by the NI derivative, which could be bio-reduced from hydrophobic to hydrophilic under hypoxic conditions, to provide hypoxia-sensitive ability. Furthermore, the crosslinking of NI and thiol groups could make the derived ALG amphiphilic, which further enabled the self-assembly formation of GR-NPs in the aqueous solution.

**Fig. 1:**
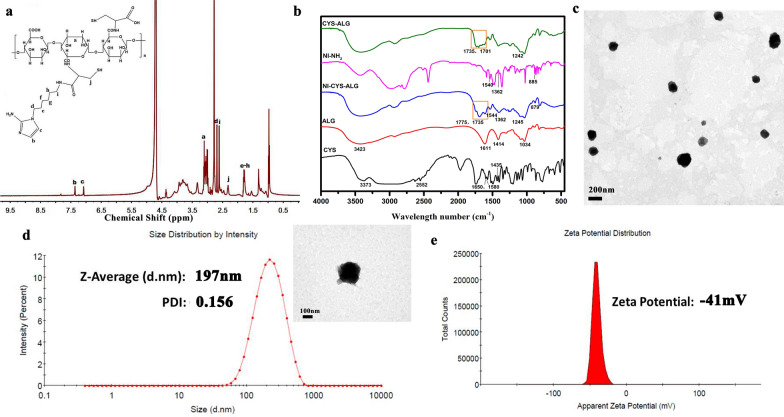
^1^H NMR spectra of NI–CYS–ALG (**a**); FTIR spectrum of NI–NH_2_, ALG, CYS, CYS–ALG, NI–CYS–ALG (**b**); TEM images of GR-NPs (**c**); particle size (**d**) and zeta potential of GR-NPs in DLS detection (**e**)

Herein, chemical structures of resultant NI derivatives, CYS–ALG and NI–CYS–ALG were further verified using ^1^H NMR analysis (details in Additional file 1: Figure S2, Fig. 1a). From the spectrum of NI–CYS–ALG conjugate (Fig. 1a), the characteristic peaks at 1.79, 2.61, 2.68, 7.08 and 7.36 ppm were attributed to the NI derivatives. The proton peaks that ranged from 3.60 to 3.93 ppm could be assigned to the ALG segments. The characteristic peaks of the CYS are shown at about 2.32 ppm [23].

The chemical composition of NI–CYS–ALG conjugates was supported by FTIR spectra analysis [24]. In Fig. 1b, the characteristic peaks of ALG are shown as follows: peaks at 1611 and 1414 cm^−1^ corresponding to asymmetric and symmetric C=O stretching, a peak at 1034 cm^−1^ (C–O–C stretch), and the broad band at 3423 cm^−1^ assigned to the stretching vibration of O–H, the intermolecular hydrogen bonds of alginate and the extension vibration of N–H. Comparing the FTIR spectra of ALG, CYS and CYS–ALG, some new peaks appeared at 1735 and 1701 cm^−1^ corresponding to the amide I and II bands, respectively. In the FTIR spectrum of NI–CYS–ALG, amide bond peaks at 1775 and 1735 cm^−1^ were enhanced and the characteristic peaks of NI derivatives (NI-NH_2_) at 1544, 1362 (asymmetric and symmetric N–O stretch) and 879 cm^−1^ (C–N stretch) appeared, indicating that NI and CYS units were successfully grafted onto ALG. The content of cysteine could improve the adhesion of NI–CYS–ALG conjugate and GR-NPs. In addition, the drug encapsulation efficiency could be enhanced with the increasing weight ratio of CYS/ALG. The concentration of CYS and NI was given in Additional file 1: Table S1. When the weight ratio of CYS/ALG was 2:1, the content of CYS and NI was 332.06 ± 6.31 μmol/g and 310.39 ± 6.59 μmol/g, respectively. The muco-adhesion of NI–CYS–ALG conjugate and GR-NPs with different weight ratios of cysteine was detected by mucin particle adhesion method (Additional file 1: Figures S3, S4). When the weight ratio of CYS/ALG was 2:1 and the conjugate concentration was 0.1%, the zeta potential value was − (11.17 ± 0.72) mV which was 6.3 times higher than that of CYS/ALG at 1:2. Moreover, with the increasing concentration of conjugate, the zeta potential value went to negative gradually. Similarly, the zeta potential value of mucin was − (17.90 ± 4.27) mV when the weight ratio of CYS/ALG of GR-NPs at 2:1, which had a significant decreasing trend than that of CYS/ALG at 1:1. Thus, the conjugates and nanoparticles play superior adhesion ability with the weight ratio of CYS/ALG in 2:1 which was the optimal formula.

As presented in the TEM image, the GR-NPs in DI-water presented a clear morphology with mono-size distribution under the power of 30 kV and 60 kV in Fig. 1c, d, respectively. The average diameter of GR-NPs was determined as 197 nm by DLS (Fig. 1d) and 160.64 ± 79.88 nm by TEM (Additional file 1: Figure S5) and zeta potential value was measured as − 41 mV (Fig. 1e), which showed the dispersion stability of GR-NPs. The insulin loading capacity and encapsulation efficiency of GR-NPs were then detected as shown in Additional file 1: Figure S6. Considering the high drug encapsulation efficiency (83.76 ± 4.20%) and good drug loading (5.49 ± 0.13%) as well as good adhesion capacity, the formula of CYS/ALG at 2:1 was selected for further experiment. The size of GR-NPs shown in Additional file 1: Figure S7 presented slightly increase from 200 to 253 nm, which were detected by DLS within different days (1 day, 3 days, 5 days).

### Insulin pH stability and enzyme inhibition studies

**Fig. 2:**
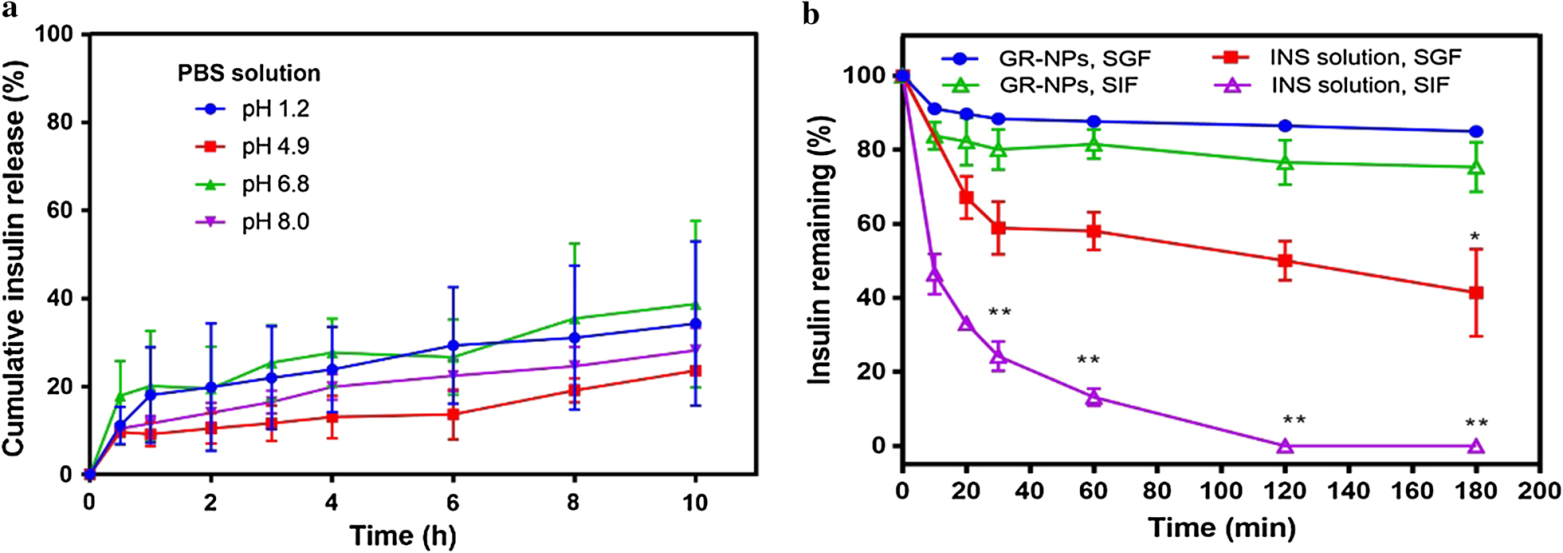
The in vitro insulin release from GR-NPs in PBS buffer with different pH values (pH 1.2, 4.9, 6.8 and 8.0) (**a**); the residual insulin amount of GR-NPs in simulated gastric fluid (SGF) and simulated intestinal fluid (SIF) buffers (**b**), a significantly higher (**P < 0.01) efficacy of the residual insulin released form GR-NPs was observed at various time points in SIF/SGF compared with pure insulin

Compared with injection, the non-invasive oral administration route is regarded as a more acceptable and easier way to improve patient compliance. Some protein drugs such as insulin in the GI tract are confronted with problems such as that drugs may have reduced stability and decreased bioavailability in gastric fluid containing hydrochloric acid and pepsin [25, 26]. To examine the pH stability of insulin-loaded GR-NPs, samples were incubated with PBS with different pH values (pH 1.2, 4.9, 6.8 and 8.0). As illustrated in Fig. 2a, there was less than 30% of cumulative insulin release when incubated in PBS with different pH levels for 4 h and less than 50% of cumulative release for 10 h from the GR-NPs, which validated the pH stability of the GR-NPs. Alginate, representing a polyanion extracted from brown algae, was chosen as hydrophilic polymer with muco-adhesive properties. Since it is reported to be non-toxic and biodegradable when given orally, and thus widely used in biomedical applications, it should not bear any toxic risks [27]. Because the membranes of self-assembly nanoparticles formed by amphiphilic polymer have a low swelling ratio under low pH, less insulin will be released [28]. As illustrated in Fig. 2b, the remaining ratio of pure insulin was dramatically decreased to 24.25 ± 4.0% in simulated intestinal fluid (SIF, pH 6.8 containing trypsin) and to 58. 89 ± 7.09% in simulated gastric fluid (SGF, pH 1.2 containing pepsin) within 30 min. Almost, insulin was completely released in SIF for 120 min, no insulin could be detected. In SGF, insulin was remained at 41.41 ± 11.81% for 180 min. In contrast, no significant amount of insulin was observed in the presence of GR-NPs, the insulin remaining was of 75.30 ± 6.78% in SIF and 84.95 ± 0.79% in SGF at 180 min, which indicating that the functional-ALG conjugate modified nanoparticles was efficient for insulin stability and enzymatic inhibition. The mechanism underlying the enzymatic inhibition of GR-NPs was reported in terms of thiol incorporation and Ca^2+^-dependent inhibition [29].

### Glucose-responsive detection of GR-NPs

Persistent glycemic control and a non-invasive system of insulin administration are key determinants of long-term exploration for diabetes therapy. Therefore, to form a feedback-controlled and closed-loop insulin-release system performed by stimuli-responsive polymers has received more and more attention in recent decades [30]. We prepared GR-NPs incubated in different PBS buffers containing different concentrations of glucose (0 mg/dL, 100 mg/dL and 400 mg/dL) to examine the glucose-responsive capability of GR-NPs. Hypoxic environment could be caused by the oxidation of glucose catalyzed by GOx according to the following catalyzed reaction:

$$Glucose + O_{2} + H_{2} O\mathop{\longrightarrow}^{GOx}Gluconic\; acid + H_{2} O_{2} .$$

**Fig. 3:**
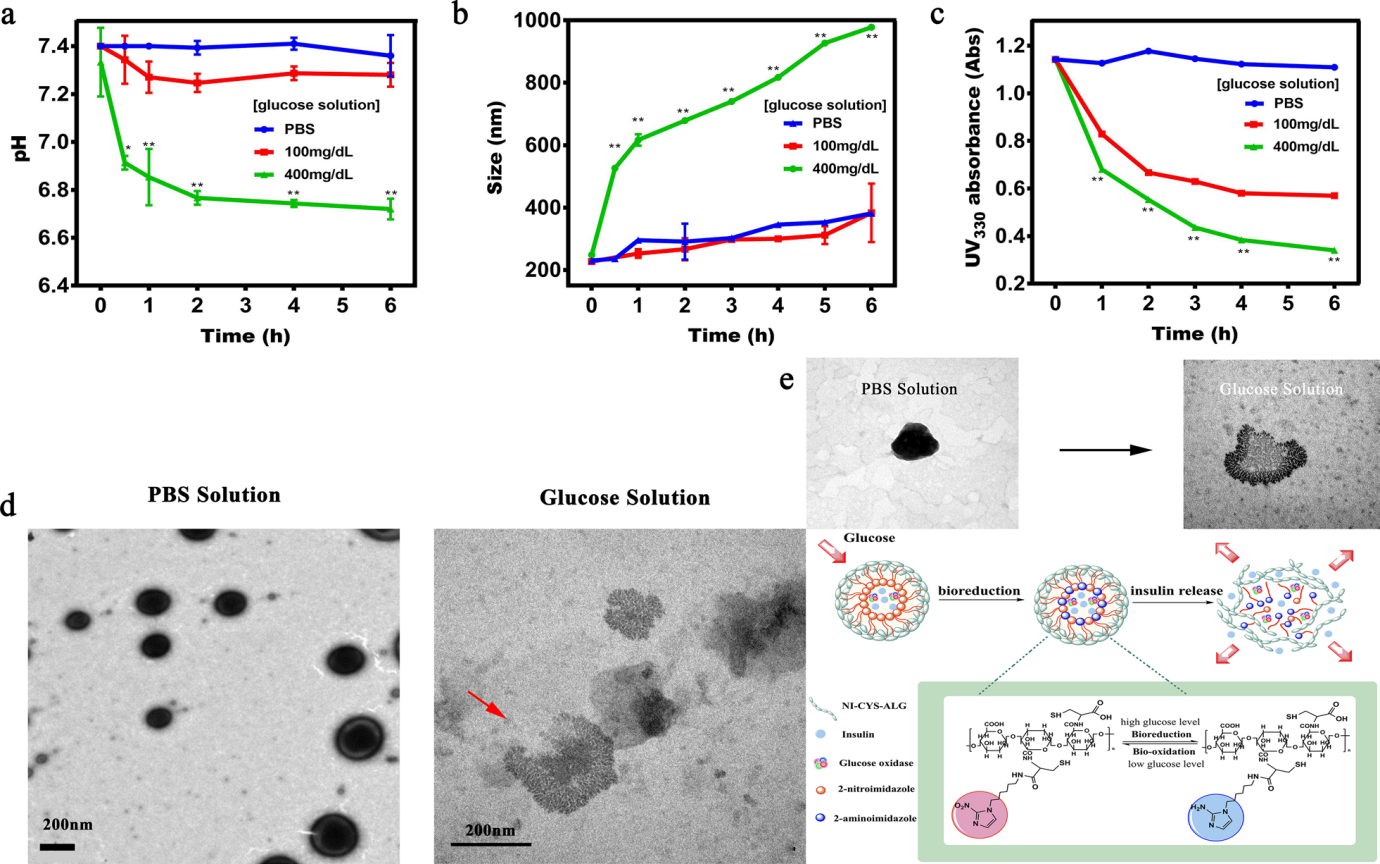
pH changes (**a**), particle size changes (**b**) and UV_330_ absorbance (**c**) of GR-NPs under different glucose concentrations (0 mg/dL, 100 mg/dL and 400 mg/dL); morphology changes of GR-NPs in PBS and glucose solution (400 mg/dL) (**d**); mechanistic diagram of GR-NPs (**e**). A significantly higher (**P < 0.01, *P < 0.05) efficacy of GR-NPs was observed at various time points in 400 mg/dL compared with in PBS buffer

As shown in Fig. 3a, compared with normoglycemic (100 mg/dL) and control (0 mg/dL) concentrations, the pH value of PBS buffers with a hyperglycemic concentration (400 mg/dL) decreased gradually from 7.4 to 6.5, which was caused by the conversion of glucose to gluconic acid catalyzed by GOx [31]. The size of GR-NPs increased from 230 to 616 nm in initial 1 h and consistently increased to 977 nm within 6 h when incubated in hyperglycemic solution, which was caused by the bioreduction of NI and was confirmed by DLS observation (Fig. 3b). In contrast, samples in the normoglycemic (100 mg/dL) and control solution groups showed slight size changes in 6 h at the range of 230 nm and 380 nm. Furthermore, the GR-NPs could undergo structural transformations (i.e. shrink, swell or dissociate) regulated by glucose concentration changes, leading to insulin release [32]. In the UV–vis spectrum (Fig. 3c), the residual concentrations of NI detected at the characteristic peak of UV_330_ were decreased when incubated in glucose solution, but no changes were observed in the control solution, which confirmed that hydrophobic nitroimidazole was replaced with hydrophilic aminoimidazole under the hypoxic environment. TEM images further illustrated the determined changes in morphology and size of GR-NPs after separately soaked in PBS and glucose solution (400 mg/dL) for 1 h (Fig. 3d). The size of GR-NPs incubated in hyperglycemic solution steadily increased over time and the morphology of GR-NPs presented changes indicating breaking-up. Nitroimidazole is one of the most hypoxic tissue sensitizers and can be reduced into an amine derivative by a series of nitroreductases in the presence of an electron donor. For this, the conversion requires an enzyme-mediated single-electron reduction of the nitro group to a free radical anion [33]. The assembly and release strategy of GR-NPs in the current report was schematically depicted in Fig. 3e.

### Intelligent insulin release from GR-NPs in vitro

**Fig. 4:**
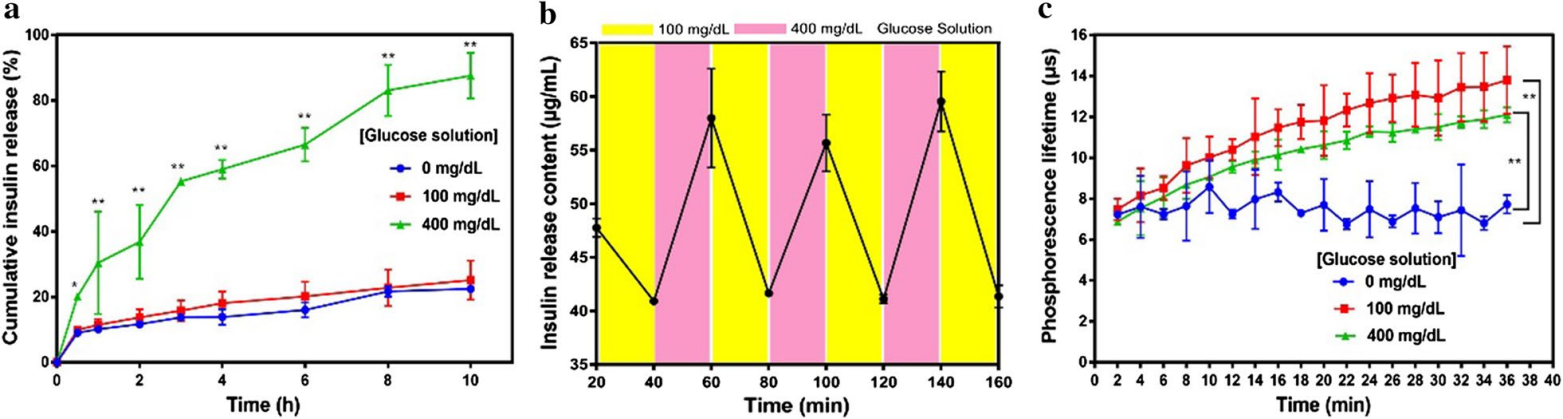
The in vitro cumulative insulin release from GR-NPs in PBS buffer with different glucose concentrations (0 mg/dL, 100 mg/dL and 400 mg/dL) (**a**); pulsatile release profile of GR-NPs presents the rate of insulin release as a function of glucose concentration (100 mg/dL and 400 mg/dL) (**b**); phosphorescence lifetime of the GR-NPs incubated with different glucose concentration solutions (0 mg/dL, 100 mg/dL and 400 mg/dL) containing an oxygen concentration molecule probe (**c**). A significantly higher (**P < 0.01) efficacy of GR-NPs was observed at various time points in 400 mg/dL compared with in PBS buffer

The glucose-responsive release profile of insulin was obviously observed in different glucose concentrations in Fig. 4a. GR-NPs exposed to hyperglycemic solution (400 mg/dL glucose) had a significant high insulin release ratio (30.43 ± 15.64% for 1 h and 87.56 ± 6.95% for 10 h), due to catalytic reaction of GOx and reverse capacity of 2-nitroimidazole resulted in the dissociation of GR-NPs. Comparatively, in normoglycemic (100 mg/dL glucose) and control solution, the insulin cumulative release was only 25.10 ± 5.97% and 22.46 ± 0.47%, respectively, which had a relatively lower release for 10 h, validating the glucose-responsiveness of this designed-NPs. In addition, it was also confirmed that GR-NPs in varying glucose concentrations presented alterable insulin release profiles (Fig. 4b). The content of insulin was detected at 55.24 ± 5.23 μg/mL after released in high glucose level, and when transferred to low glucose level the content was 41.26 ± 0.31 μg/mL, showing an alterable kinetic release ability. As illustrated in Fig. 4c, the GR-NPs exposed to the hyperglycemic solution (400 mg/dL glucose) had a lower oxygen concentration compared with the other two control samples (PBS and 100 mg/dL), which further confirmed the hypoxia-sensitive of GR-NPs when exposed in hyperglycemic solution.

### Biocompatibility and chemical stability of insulin

**Fig. 5:**
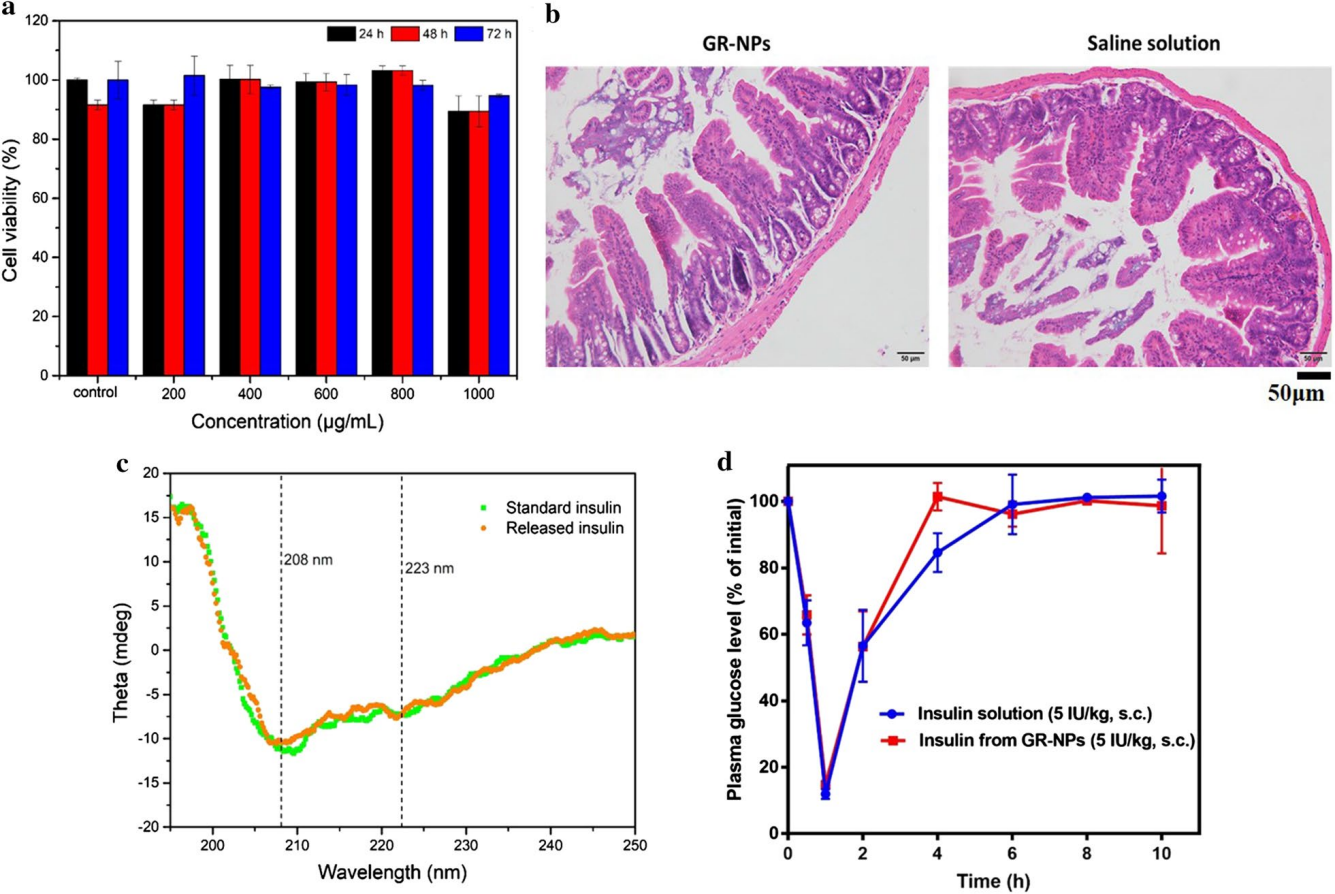
The viability of Caco-2 cells in different GR-NPs concentrations (0 to 1000 μg/mL) cultured for different times (24 h, 48 h or 72 h) (**a**); H&E staining of normal mice intestine after oral administration of GR-NPs and saline for 3 h (**b**); CD spectroscopy (**c**) and hypoglycemic activity in mice of standard insulin and insulin released from GR-NPs (**d**)

Figure 5a showed the cell viability of Caco-2 cells treated with GR-NPs using a MTT assay with different concentrations of GR-NPs after incubating for 24 h, 48 h or 72 h. The cells maintained > 90% viability when the concentrations ranged from 0 to 1000 μg/mL, indicating good biological safety of the GR-NPs. H&E staining of normal mice following oral administration of 1 mL of GR-NPs and saline for 3 h confirmed the good biocompatibility of GR-NPs in vivo, which illustrated no significant tissue lesions (Fig. 5b). The good biocompatibility of GR-NPs on SD rats was also detected in Additional file 1: Figure S8 by H&E staining. The results of cell cytotoxicity and tissue lesions showed that, as designed, GR-NPs possessed excellent cytocompatibility and, therefore, had great potential for oral application.

To evaluate the chemical stability of insulin released from GR-NPs, an effective technique of CD spectroscopy was applied to evaluate the conformational stability of proteins [34]. As presented in Fig. 5c, far-UV CD spectroscopy of the insulin released from NPs and pure insulin solution showed two negative bands at 208 nm and 223 nm, which were typical and corresponded to the predominant α-helix structure and β-pleated sheet structure, respectively. The ratio of intensity of 208 and 223 nm bands ([Ф]_208_/[Ф]_223_) has usually been employed to provide a qualitative measure of insulin association. The [Ф]_208_/[Ф]_223_ ratios of native and released insulin from GR-NPs were 1.661 and 1.751, respectively, which reflected that there was no significant difference in the secondary structure between the native and released insulin.

Bioactivity of insulin in GR-NPs was further investigated after subcutaneous injection of insulin released from destroyed GR-NPs. The blood glucose level of mice was exhibited to be similar to during hypoglycemic activity, there was no significant difference between the hypoglycemic effects of insulin and that of the insulin released from GR-NPs (Fig. 5d). It was concluded that the bioactivity of insulin was well preserved in nanoparticle formulations. The hypoglycemic effect of insulin loaded in GR-NPs via oral management could further confirm the bioactivity of insulin presented in Fig. 7a.

### Muco-adhesion studies of GR-NPs

Bioadhesive systems such as adhesion promoter PEG, cationic polymers like chitosan, modified lectin-copolymer like ConA and so on usually provide only weak adhesion based on the formation of non-covalent bonds such as van der Waal’s force, hydrogen bonds and ionic interactions [35–37]. Thus, in many cases these approaches are insufficient to guarantee the location of a drug delivery system at a given target site. Muco-adhesive polymers, i.e. thiolated polymer, because of covalent disulfide bonds with thiol bearing mucus substructures are capable of forming covalent bonds by interacting with cysteine-rich subdomains of mucus glycoproteins that should provide a prolonged residence time of delivery systems on epithelium [38]. We measured the adhesive interaction of GR-NPs to mucin by using the changes in zeta potential of the original mucin particles after mixing with GR-NPs via mucin-particle method (detail in Additional file 1) [39]. Thiolated polymers, or so-called thiol polymers, can avoid adhesion failure of the muco-adhesive polymer itself, owing to their high cohesive properties. As presented in Additional file 1: Figure S3, the addition of thiol groups in NI–CYS–ALG conjugates led to the more extensive changes in zeta potential. Additionally, thiol groups in GR-NPs could interact with thiol-rich subdomains of mucus, which caused the resulted zeta potential of mucin shifted from 0 mV to an extensively negative value (about − 20 mV) as shown in Additional file 1: Figure S4.

**Fig. 6:**
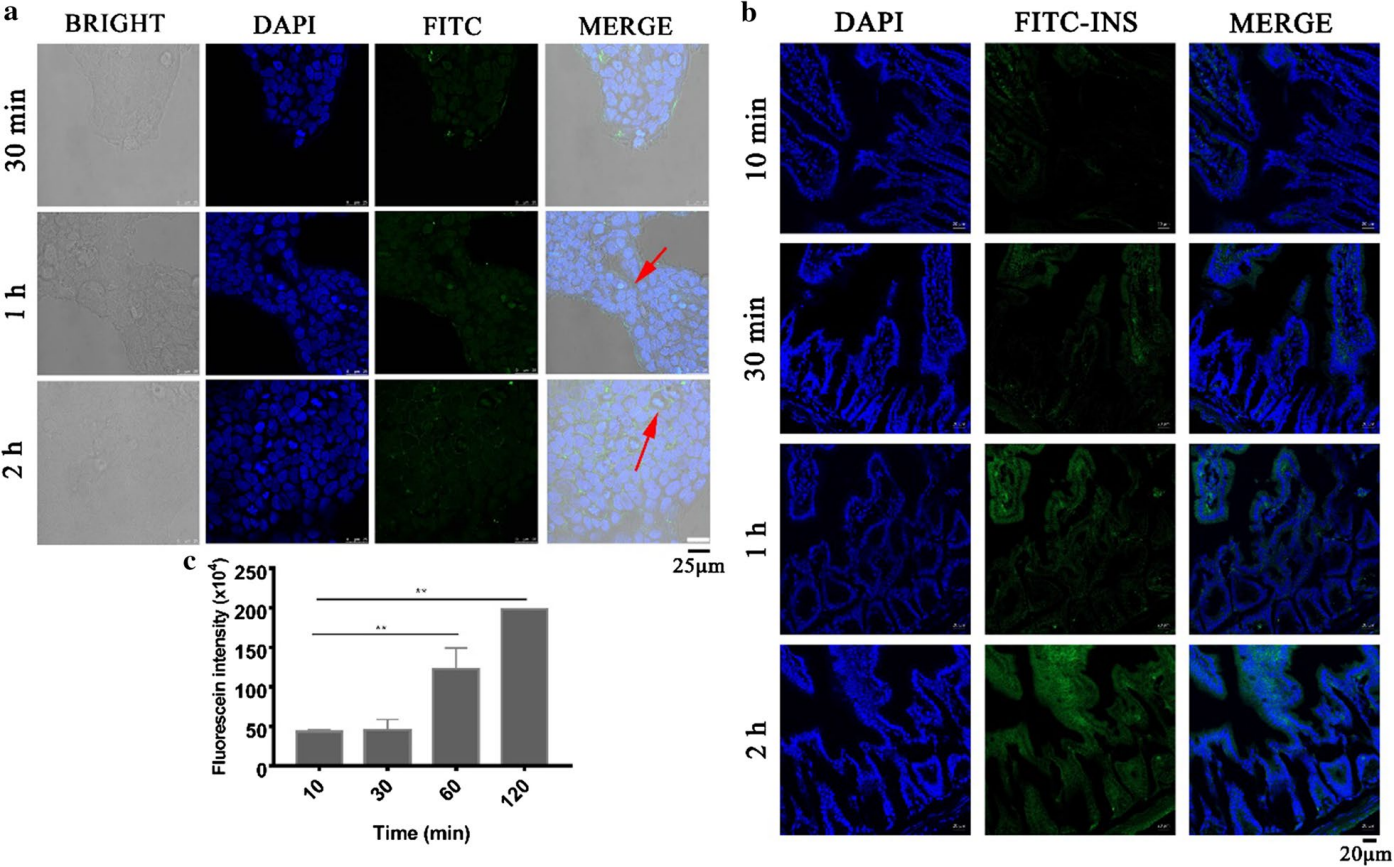
CLSM images of the adhesion and endocytosis of FITC-INS/GOx loaded GR-NPs in Caco-2 cells in different time (30 min, 1 h, 2 h) (**a**); The CLSM images (**b**) and fluorescein intensity (**c**) of FITC-INS/GOx loaded GR-NPs in intestinum tenue of mice after 10 min, 30 min, 1 h and 2 h oral administration (represented as **P < 0.01)

The biological utilization of therapeutic proteins and peptides by the oral route is mostly lacking due to gastric acid degradation, enzyme degradation, large molecular size and poor permeation [40]. With focus on technological trends to improve the bioavailability of therapeutic proteins and peptides by the oral route, chemical transformation of protein structures, enzyme inhibitors, muco-adhesive polymers and permeation enhancers are currently used as effective oral strategies [41, 42]. Besides, it was reported that the intestinal uptake of therapeutic proteins through adopted nanoparticles regulated by different particle sizes can improve the transporting ability of proteins through the epithelial lining of the small intestine while protecting the proteins against degradation in gastric fluid [43]. Cell adhesive and penetrating properties of the insulin-loaded GR-NPs were evaluated by CLSM after different incubation times (30 min, 1 h or 2 h) with Caco-2 cells. As presented in Additional file 1: Figure S9, cells were counterstained with DAPI (blue) and insulin was labeled by FITC (green) and membrane of cells was stained with TRITC Phalloidin (red), the green fluorescence could be observed after 30 min incubation showing a good adhesion of GR-NPs at the cell surface, while no signal was seen in control group (FITC-INS). In Fig. 6a, the green fluorescence appeared in early 30 min and could still existed in 2 h, indicating a certain extent of adhesion and uptake of FITC-INS/GOx loaded GR-NPs at the cell surface [44].

In order to identify the bioadhesion of GR-NPs at the location of small intestine, the FITC-INS/GOx loaded GR-NPs were orally administered to diabetic rat. At specific time intervals (10 min, 30 min, 1 h or 2 h), mice were sacrificed and the GI tract was exposed to determine the absorption of the FITC-INS/GOx loaded GR-NPs in GI-tract. As shown in Fig. 6b, weak green fluorescence should be seen after 10 min oral administration and got stronger with increasing time, a significant increase in signal 2 h later (Fig. 6c). The fluorescence intensity of 60 min and 120 min was 2.8 times and 4.5 times higher than that of 30 min, respectively. This result was consistent with CLSM observation of Caco-2 cells in vitro (Fig. 6a). As shown in Additional file 1: Figure S10, the bioadhesion ability of FITC-INS/GOx loaded GR-NPs on duodenum, ileum, jejunum and colon of SD rats was significantly observed after 2 h oral administration. In contrast, the signal of pure FITC-INS showed a weak green fluorescence in different parts of small intestine. It is confirmed that the GR-NPs can prolong the residence time of FITC-INS to intestine.

### Blood glucose regulation

**Fig. 7:**
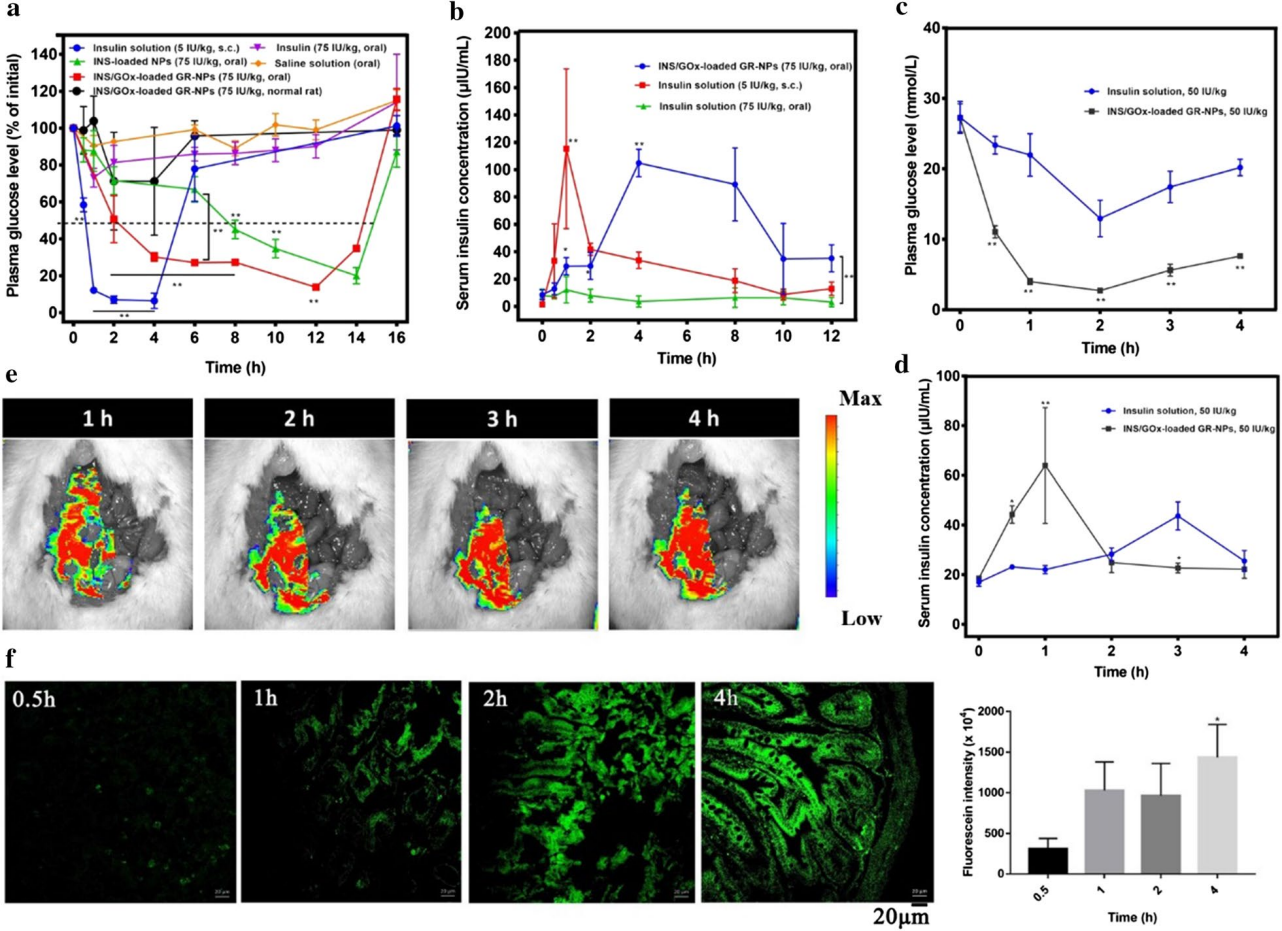
Plasma glucose levels in diabetic rats following oral administration of INS-loaded NPs, INS/GOx GR-NPs, saline or insulin solution. Intraperitoneal injection of insulin solution and in normal rats following oral administration of INS/GOx GR-NPs (**a**); serum insulin concentration in diabetic rats following oral administration of INS/GOx GR-NPs and insulin solution, intraperitoneal injection of insulin solution (**b**), *P < 0.05, **P < 0.01: significance: comparison between oral GR-NPs, INS-loaded NPs and s.c. administration insulin; plasma glucose levels in diabetic rats following ileal administration of INS/GOx GR-NPs and insulin solution (**c**); serum insulin concentration in diabetic rats following ileal administration of INS/GOx GR-NPs and insulin solution (**d**), *P < 0.05, **P < 0.01: significance: comparison between INS-loaded NPs and insulin; ex vivo living scanning image of rats after administration of FITC-INS/GOx GR-NPs for different times (1 h, 2 h, 3 h or 4 h) (**e**); CLSM images and fluorescence intensity of FITC-INS/GOx GR-NPs in intestinal tissue of rat after 0.5 h, 1 h, 2 h or 4 h oral administration (**f**)

Figure 7a showed the behavior of different formulations administered to diabetic rats. Almost no hypoglycemic effect could be created by oral administration of free insulin. Both GR-NPs (with INS and GOx) and INS-loaded NPs exhibited relatively strong hypoglycemic effects compared with subcutaneous injection of insulin solution after 6 h administration (**P < 0.01). The blood glucose level of pure insulin decreased to 12.2 ± 1.0% of the initial level by 1 h, and returned to the hyperglycemia level after 4 h. However, after oral administration of INS-loaded NPs (75 IU/kg) the blood glucose level decreased to 45.1 ± 4.1% of initial concentration by 8 h and could maintain the range of euglycemic levels for 6 h. Compared with INS-loaded NPs, GR-NPs (75 IU/kg) presented a better hypoglycemic effect (**P < 0.01), which decreased the blood glucose from initial to 30.2 ± 1.9% for 2 h and could maintain euglycemic levels up to 12 h. These results indicate a sustained drug release of INS-loaded NPs by oral administration, more importantly GR-NPs exhibited a significant hypoglycemic effect because of its effective catalysis of blood glucose, which could cause a hypoxic environment that could cause 2-nitroimidazole to be bioreduced from hydrophobic to hydrophilic. Of note, no significant hypoglycemic effect could be seen by the oral administration of GR-NPs in normal rats, and presented no hypoglycemia in rats that possessed regular self-administration. The hypoglycemic effect on diabetes mice was also detected, illustrating similar blood glucose regulation (Additional file 1: Figure S11).

Serum insulin level analysis was further investigated to determine the relative bioavailability of the released insulin. Figure 7b shows the serum insulin concentration reached the peak value at 111.5 μIU/mL after administration by intraperitoneal injection of insulin solution for a short time and then decreased quickly to a lower level. However, the serum insulin concentration increased to 104.5 μIU/mL after oral administration of GR-NPs after 4 h and could maintain the retention time at a high level for 4 h. In contrast, there was no change in insulin concentration after oral insulin administration, showing better bioavailability of GR-NPs.

Comparing insulin solution (50 IU/kg) and GR-NPs (50 IU/kg) in rats by ileal administration, the plasma blood glucose value was decreased from 28 to 15 mmol/L within 2 h and then increased gradually after administrated by GR-NPs (50 IU/kg), as shown in Fig. 7c, presented a better hypoglycemic effect after 4 h than INS solution, which was restricted by barriers of absorption and enzymatic degradation in ileum. Similarly, Fig. 7d shows significant bioavailability of GR-NPs, which reached the peak value of serum insulin concentration at 52.1 μIU/mL for 1 h, indicating the adhesion and good penetrating of GR-NPs in ileum via thiol group could improve insulin absorption.

In order to evaluate the GI tract absorption and deposition of the GR-NPs, FITC-INS/GOx loaded GR-NPs were delivered to rat by oral administration and observed by live imaging. As presented in Fig. 7e, a strong luminescent signal could be observed in GI tract. Subsequently, the luminescent signal could still be detected in the intestinal tract for 4 h, indicating that FITC-INS/GOx loaded GR-NPs could be successfully delivered to the intestinal tract and adhere to intestinal epithelial cells, promoting drug absorption. Additionally, after 0.5 h, 1 h, 2 h or 4 h of oral administration of FITC-INS/GOx loaded GR-NPs, the animals were sacrificed and the small intestinal was removed to be observed by CLSM. In Fig. 7f, it can be seen that fluorescent signal was enhanced over time, which was performed for the confirmation of deposition of the GR-NPs in the GI tract.

### Mechanism of GR-NP transport

**Fig. 8:**
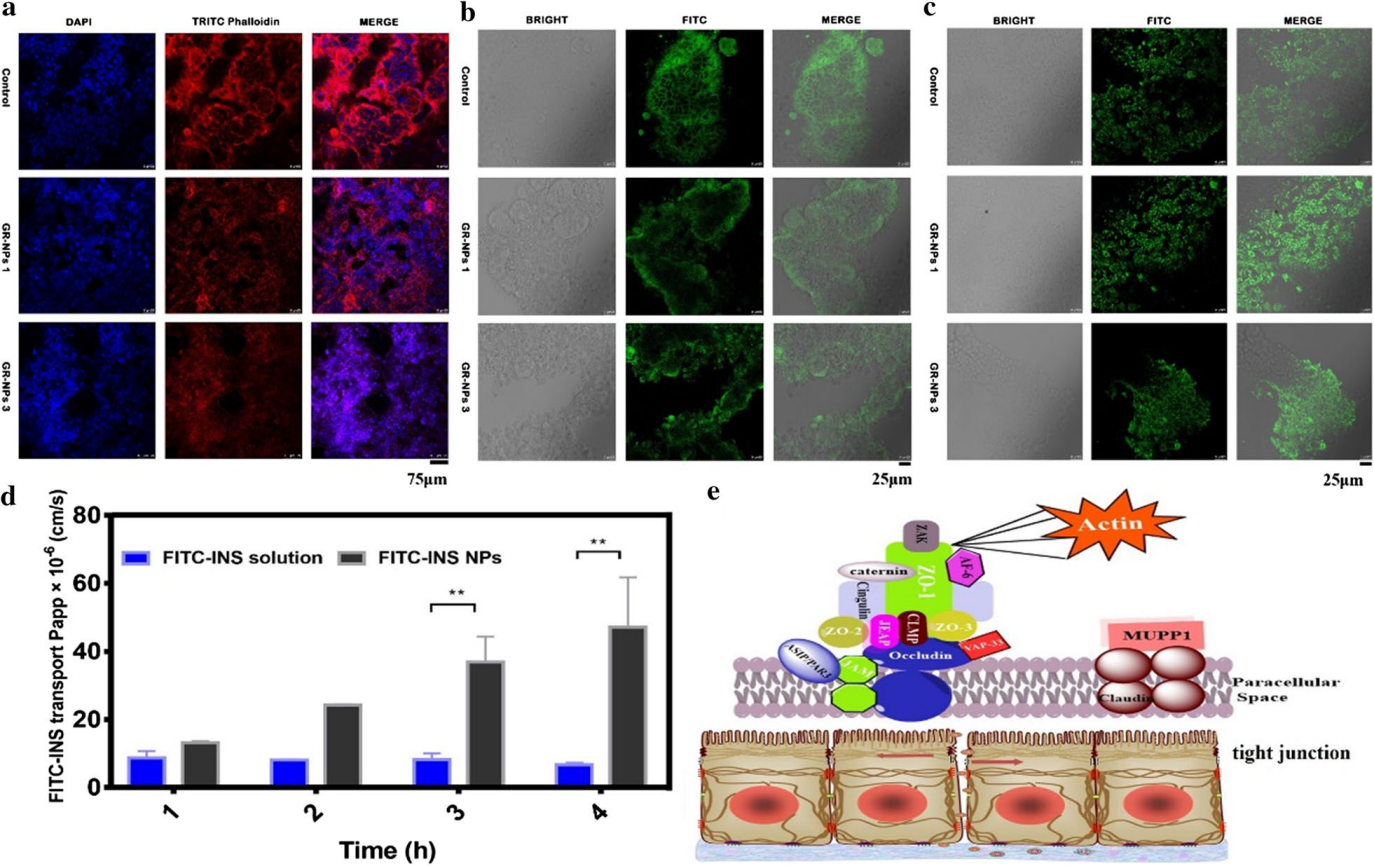
CLSM observation of the distribution of F-actin (**a**), ZO-1 (**b**) and occludin (**c**) on Caco-2 cells cultured with GR-NPs 1 and GR-NPs 3; The transport permeability of FITC-INS and FITC-INS/GOx GR-NPs in fresh intestine (**d**), **P < 0.01: significance: comparison between FITC-INS/GOx GR-NPs and FITC-INS solution; Schematic illustration of GR-NPs overcoming epithelial barriers on intestinal cell (**e**)

Tight junctions (TJs) are one of the main types of intercellular junctions existing in GI epithelial cells. In order to detect transcellular permeation, Caco-2 epithelial cell monolayers are commonly used to explore the attachment of the drug delivery system and tight junctions in vitro [45]. As shown in Fig. 8a, cytoskeletal F-actin was normally distributed in control (insulin) administrated Caco-2 cells while GR-NPs could make cytoskeletal F-actin discontinuous. In comparison, GR-NPs 3 led to significant discontinuous F-actin in Caco-2 cells for 2 h than GR-NPs 1, which may be caused by the impact of cysteine. Protein kinase C (PKC) is an important part of tight junction function, inducing the distribution of ZO-1 (a bridge protein) and occludin moving between cell membranes that could directly affect the structure and function of TJs (Fig. 8e). In this paper, the effect of GR-NPs on tight junctions was investigated with ZO-1 and occludin. As shown in Fig. 8b, compared with the control group (insulin), the distribution of ZO-1 in Caco-2 cells treated with both GR-NPs 1 and GR-NPs 3 for 2 h was changed, i.e. discontinuous, which could illustrate the impact of the structure of tight junctions. However, the distribution of occludin on Caco-2 cells, shown in Fig. 8c, had no change after administration of GR-NPs, which indicated the paracellular penetrating of GR-NPs might through the interaction between GR-NPs and membrane via F-actin and ZO-1 and other related proteins.

Protein drugs like insulin have a lack of lipophilicity, no passive absorption and are absorbed primarily through paracellular pathways, hence large numbers of transport mechanisms such as simple diffusion (paracellular and transcellular), carrier-mediated transport, active transport and pinocytosis or endocytosis are responsible for penetration. The paracellular route is not feasible for large macromolecules, of which the space lies between 10 and 30–50 Å. Transportation of drugs (e.g. insulin) across the oral mucosa is regulated by molecular geometry, lipophilicity and charge of the transport pathway [46, 47]. In order to validate the improving intestinal permeability of insulin from the GR-NPs, insulin conjugated with FITC was encapsulated into GR-NPs. As demonstrated in Fig. 8d, FITC-INS/GOx loaded GR-NPs showed a great transport capacity, at the range from 13.19 ± 0.32 to 47.15 ± 10.30 (× 10^−6^ cm/s), after encapsulated to intestinal tissue (both ends were binding) and then exposed it to Krebs–Ringer for different times (1 h, 2 h, 3 h, 4 h), while the pure FITC-INS showed a low apparent permeability coefficient value (Papp) between 6.74 ± 0.36 to 8.6 ± 1.43 (× 10^−6^ cm/s) verifying the good intestinal permeability of GR-NPs. Altogether, GR-NPs can open tight junctions by inducing changes of cytoskeletal F-actin and ZO-1 redistribution that can further improve transcellular permeability [48].

## Conclusion

In this study, we describe an original method for fabrication of a glucose-responsive nanoparticle system loaded with insulin and glucose oxidase (GOx) enzyme that self-assembles by amphipathic 2-nitroimidazole-l-cysteine-alginate (NI–CYS–ALG). The superior weight ratio of CYS/ALG in GR-NPs is 2:1, showing good bioadhesion and encapsulation efficiency, and also presenting a good pH stability and anti GI tract enzyme possibility, which is good for oral delivery. In vitro tests illustrated that the insulin release behavior is switched “*ON*” in response to hyperglycemic state by GOx catalysis and “*OFF*” by normal glucose levels. In addition, in vivo tests on type I diabetic rat displayed a significant hypoglycemic effect, avoiding the risk of hyperglycemia and maintaining a normal range for a relatively long time after oral administration. Moreover, the mechanism of GR-NPs transport through epithelial cells may be caused by the high content of cysteine on GR-NPs.

To summarize, we have shown that:

1. It is possible to achieve self-assembly of nanoparticles by amphiphilic alginate via modification with functional 2-nitroimidazole and l-cysteine. Undergo structural transformations of GR-NPs adjusted by glucose concentration changes, leading to glucose-stimulated insulin release under the hypoxic environment catalyzed by GOx.
2. The purpose of the study was to improve the muco-adhesive properties of alginate by the covalent attachment of cysteine, which can improve intestinal permeability. These features should render thiolated alginate useful as an excipient for various drug delivery systems, providing improved stability and prolonged residence time on certain mucosal epithelia.
3. The GR-NPs system is an effective and safe peroral carrier for protein drugs that can overcome pH and enzyme degradation.
4. More importantly, the described work is of a synthetic glucose-responsive nanoparticle system using a hypoxia trigger for regulation of insulin release, which holds the promise of avoiding the risk of hyperglycemia and hypoglycemia for diabetes therapy.

## Supplementary information

**Additional file 1: Experiment methods.** Synthesis of NI derivative (NI-BOC, NI-NH_2_), CYS-ALG and NI-CYS-ALG polymers; degree detection of NI and CYS; content detection of insulin; muco-adhesion studies of NI-CYS-ALG and GR-NPs; hypoglycemic effect of GR-NPs on mice. **Figure S1.** The synthetic flow chart of NI-CYS-ALG conjugate. **Figure S2.**^1^H NMR spectrum of 2-nitroimidazole, 6-(boc-amino)hexyl bromide, NI derivative (NI-BOC, NI-NH_2_), CYS, ALG and CYS-ALG. **Figure S3.** Zeta potential changes of NI-CYS-ALG conjugate under mucin particle adhesion method with different contents of cysteine (1:2-2:1, weight ratios). **Figure S4.** Zeta potential changes of GR-NPs under mucin particle adhesion method with different contents of cysteine (1:2, 1:1, 2:1, weight ratios). **Figure S5.** TEM images and size distribution of GR-NPs. **Figure S6.** Drug loading and encapsulation efficiency of GR-NPs with different content of cysteine (1:2, 1:1, 2:1, weight ratios). **Figure S7.** Particle size of GR-NPs in different days (1d, 3d, 5d). **Figure S8.** H&E staining observation of SD rat intestine after oral administration of GR-NPs and saline for 3 h. **Figure S9.** CLSM observation of cell adhesion with FITC-INS (control) and FITC-INS/GOx loaded GR-NPs after 30 min incubation. **Figure S10.** CLSM images of FITC-INS/GOx GR-NPs and FITC-INS in intestinal tissue (duodenum, ileum, jejunum and colon) of rats after 2 h oral administration. **Figure S11.** Plasma glucose levels in diabetic mice following oral administration of INS-loaded NPs, INS/GOx GR-NPs, saline or insulin solution, and following subcutaneous injection of insulin solution, and in normal rats following oral administration of INS/GOx GR-NPs. **Table S1.** Concentration of CYS and NI in NI-CYS-ALG polymer with different weight ratios of CYS/ALG. **Table S2.** Particle size and Zeta potential of GR-NPs.

## Data Availability

All data generated or analysed during this study are included in this article and its additional file.

## Content for publication

All authors agreed to submit this manuscript.
