# Supplementary material for: Oral delivery of insulin with intelligent glucose-responsive switch for blood glucose regulation
Source: J Nanobiotechnology. 2020 Jul 14;18:96. doi: 10.1186/s12951-020-00652-z (PMC7362448; doi:10.1186/s12951-020-00652-z)
Supplement: Supplementary file 1 — Additional file 1: Experiment methods. Synthesis of NI derivative (NI-BOC, NI-NH2), CYS-ALG and NI-CYS-ALG polymers; degree detection of NI and CYS; content detection of insulin; muco-adhesion studies of NI-CYS-ALG and GR-NPs; hypoglycemic effect of GR-NPs on mice. Figure S1. The synthetic flow chart of NI-CYS-ALG conjugate. Figure S2.1H NMR spectrum of 2-nitroimidazole, 6-(boc-amino)hexyl bromide, NI derivative (NI-BOC, NI-NH2), CYS, ALG and CYS-ALG. Figure S3. Zeta potential changes of NI-CYS-ALG conjugate under mucin particle adhesion method with different contents of cysteine (1:2-2:1, weight ratios). Figure S4. Zeta potential changes of GR-NPs under mucin particle adhesion method with different contents of cysteine (1:2, 1:1, 2:1, weight ratios). Figure S5. TEM images and size distribution of GR-NPs. Figure S6. Drug loading and encapsulation efficiency of GR-NPs with different content of cysteine (1:2, 1:1, 2:1, weight ratios). Figure S7. Particle size of GR-NPs in different days (1d, 3d, 5d). Figure S8. H&E staining observation of SD rat intestine after oral administration of GR-NPs and saline for 3 h. Figure S9. CLSM observation of cell adhesion with FITC-INS (control) and FITC-INS/GOx loaded GR-NPs after 30 min incubation. Figure S10. CLSM images of FITC-INS/GOx GR-NPs and FITC-INS in intestinal tissue (duodenum, ileum, jejunum and colon) of rats after 2 h oral administration. Figure S11. Plasma glucose levels in diabetic mice following oral administration of INS-loaded NPs, INS/GOx GR-NPs, saline or insulin solution, and following subcutaneous injection of insulin solution, and in normal rats following oral administration of INS/GOx GR-NPs. Table S1. Concentration of CYS and NI in NI-CYS-ALG polymer with different weight ratios of CYS/ALG. Table S2. Particle size and Zeta potential of GR-NPs. [file 12951_2020_652_MOESM1_ESM.docx]

**Oral Delivery of Insulin with Intelligent Glucose-responsive Switch for Blood Glucose Regulation**

Xia Zhou^1^, Hongwei Wu^2^, Ruimin Long^1,4^, Shibin Wang^1,3,4^, Haiwang Huang^5^, Yanhua Xia^5^, Pei Wang^1^, Yifeng Lei^6^, Yuanyuan Cai^1^, Duanhua Cai^1^, Yuangang Liu^1,3,4,*^

1. College of Chemical Engineering, Huaqiao University, Xiamen 361021, China
2. Department of Chemistry, Xinxiang Medical University, Xinxiang, Henan 453003, China
3. Institute of Pharmaceutical Engineering, Huaqiao University, Xiamen 361021, China
4. Fujian Provincial Key Laboratory of Biochemical Technology, Huaqiao University, Xiamen 361021, China
5. Internal Medicine Department, Xiamen Haicang Hospital, Xiamen, Fujian 361000, China
6. The Institute of Technological Sciences & School of Power and Mechanical Engineering, Wuhan University, Wuhan 430072, China

* Correspondence: E-Mail: ygliu@hqu.edu.cn; Tel./Fax: +86-592-616-2326.

**Additional file**

**Oral Delivery of Insulin with Intelligent Glucose-responsive Switch for Blood Glucose Regulation**

**Synthesis and Characterizations of NI Derivative**

In order to prepare the amphiphilic conjugate that can form GR-NPs in an aqueous condition, the NI derivative was chemically conjugated to the back bone of water-soluble CYS-ALG through amide formation (Figure S1). In brief, NI (0.5 mmoL) was dissolved in 5 mL dimethylformamide (DMF), to which K_2_CO_3_ (0.6 mmoL) and 6-(Boc-amino)hexyl bromide (0.5 mmoL) in 1 mL DMF were added. The reaction mixture was stirred at 80 ℃ for 1 h, then cooled to room temperature, later filtered through methanol and washed by ethyl acetate (20 mL x 3), after which the residual solvent was evaporated. The residual was purified by column chromatography using PE/EtOAc to achieve tert-butyl (5-(2-nitro-1H-imidazol-1-yl)pentyl)-carbamate (productivity, 85%) [1]. **Compound 1 (NI-BOC):** ^1^H NMR (500 MHz, CDCl_3_) δ 7.91 (s, 1H), 7.06 (d, *J* = 23.4 Hz, 1H), 4.91–4.01 (m, 2H), 3.31 (t, *J* = 6.8 Hz, 1H), 3.02–2.31 (m, 8H), 1.88–1.59 (m, 2H), 1.34 (s, 9H).

The obtained compound 1 was dissolved in 10 mL methanol and cooled to 0 ℃, to which 10 mL of 1.2 N HCl in methanol was added and stirred overnight. The solvent was removed from the reaction mixture using a rotary evaporator. The crude solid was washed with dichloromethane to obtain amine-functionalized 2-nitroimidazole (productivity, 97%).

**Compound 2 (NI-NH_2_)**: ^1^H NMR (500 MHz, CDCl_3_) δ 8.26 (s, 1H), 5.32 (s, 1H), 3.44 (t, *J* = 6.7 Hz, 1H), 3.04 (s, 1H), 1.94–1.78 (m, 1H), 1.57–1.44 (m, 1H), 1.36–1.25 (m, 1H), 0.90 (t, *J* = 6.9 Hz, 1H).

**Synthesis of CYS-ALG**

Covalent attachment of L-cysteine to alginate was achieved by the formation of amide bonds between the primary amino group of the amino acid and a carboxylic acid group of the polymer [2]. 1-ethyl-3-(3-dimethylaminopropyl)carbodiimide hydrochloride (EDC) in a final concentration of 50 mM with pH at the range of 4.0-4.5 was added into 20 mL 1% alginate, which could activate the carboxylic acid moieties of alginate sodium. L-cysteine monohydrate hydrochloride (weight ratio of CYS/ALG was 1:2, 1:1, 2:1, respectively) was added and the reaction was allowed to proceed for 2 h with the pH at 4.0, later adjusted to pH 6.0 and stirred for an additional hour. The resulted CYS-ALG conjugate was isolated by dialyzing against 1 mM HCl, followed by two times against the same medium but also including 1% NaCl and, finally, exhaustively against 1 mM HCl (pH 4.0). Samples were lyophilized and then stored at 4 ℃ until further use.

**Synthesis of NI-CYS-ALG Polymers**

Next, the NI derivative was conjugated to Cys-alginate in the presence of EDC and NHS. In brief, Cys-alginate (0.1 g) was dissolved in a 1:1 mixture of formamide and dimethyl formamide (5mL), after which EDC and NHS were added and stirred for 15 min. The NI derivative (0.1 g) in 1 mL DMF was slowly added to the reaction mixture and stirred for 1 day. The resulting solution was dialyzed against an excess of water/methanol (1/1-1/3, *v/v*) for 1 day and against distilled water for 2 days before being lyophilized. Samples were lyophilized and then stored at 4 ℃ until further use.

**Detection of Degree of NI and CYS**

The thiol concentration on the NI-CYS-ALG was determined by 2,4,6-trinitrobenzenesulfonic acid solution (TNBS) and 5,5'-dithiobis-(2-nitrobenzoic acid) (DTNB) method [3]. The amount of NI derivative to CYS-ALG was spectrophotometrically determined from the characteristic peak of the NI derivative at 330 nm using a UV-vis spectrophotometer (Table S1).

**Detection of Insulin Content by HPLC method**

Freeze-dried GR-NPs (10 mg) were accurately weighed, then were suspended in 0.01 M HCl solution (1 mL) and sonicated for 5 min. The collection solution was further diluted with PBS buffer (pH 7.4) to 10 mL, and centrifuged for 20 min at 10000 rpm under 4 ℃. The supernatant was analyzed by HPLC method.

**Muco-adhesion Studies with Mucin**

The 1% mucin suspension from porcine intestinal mucosa was dissolved in pH 6.8 PBS, which was treated by ultrasonic probe for 10 min at 0 ℃ after stirring for 10 min and then centrifuged for 15 min at 5000 rpm. After centrifugation, the supernatant was diluted to 0.5%. Later, the appropriate amount of NI-CYS-ALG conjugate and GR-NPs dissolved in PBS solution with pH 6.8 was added into 0.5% mucin suspension and cultured for 2 h. The zeta potential value changes of conjugates and GR-NPs with different cysteine contents were measured by laser particle size and zeta potential analyzer (Figure S4, Figure S6) [4].

**Hypoglycemic Effect on Mice**

Healthy male ICR mice were randomly divided into five groups (five mice per group) after fasting for 12 hours before the experiment. Diabetic mice (>12.6 mmol/L) were induced by the same proposal of rats and the blood glucose level was determined using a glucose meter. Insulin/GOx loaded GR-NPs (50 IU/kg), insulin solution (50 IU/kg) and saline solution were orally delivered to diabetes mice. In comparison, insulin/GOx loaded GR-NPs (50 IU/kg) was delivered to normal mice via oral route. Insulin solution (5 IU/kg) delivered by subcutaneous injection was the control. As shown in Figure S11, the blood glucose level of pure insulin (5 IU/kg, subcutaneous injection) decreased to 14.9 ± 3.8% of the initial level by 1 h, and returned to the hyperglycemia level after 2 h. After oral administration of INS/GOx loaded GR-NPs (50 IU/kg) the blood glucose level decreased to 40.3 ± 10.8% of initial concentration by 6 h and could maintain the range of euglycemic levels for 18 h. Compared with INS/GOx-loaded GR-NPs in normal rats which presented a slight decreation (>50.33 ± 0.1% of initial), INS/GOx-loaded GR-NPs (5 IU/kg) presented a better hypoglycemic effect. Moreover, no significant hypoglycemic effect could be seen by the oral administration of insulin solution and saline solution (Figure S11) [5-7].


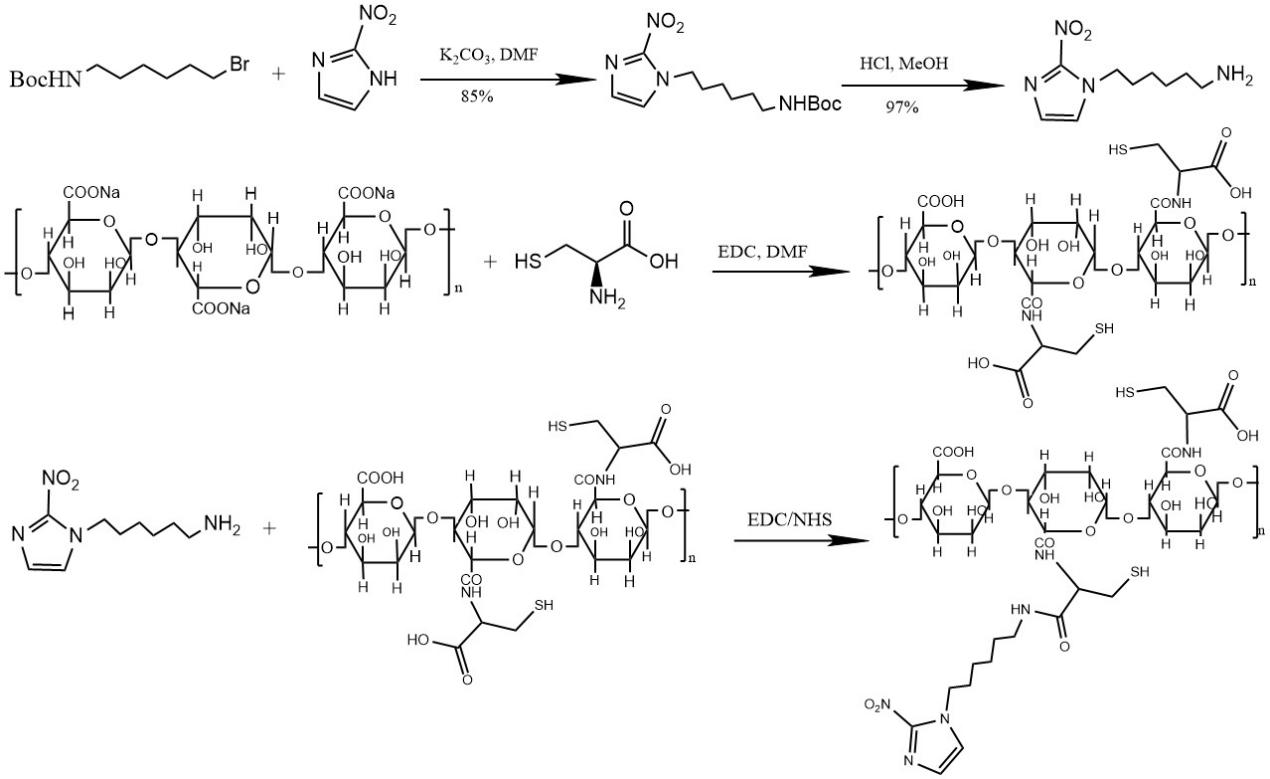


Figure S1 The synthetic flow chart of NI-CYS-ALG conjugate.


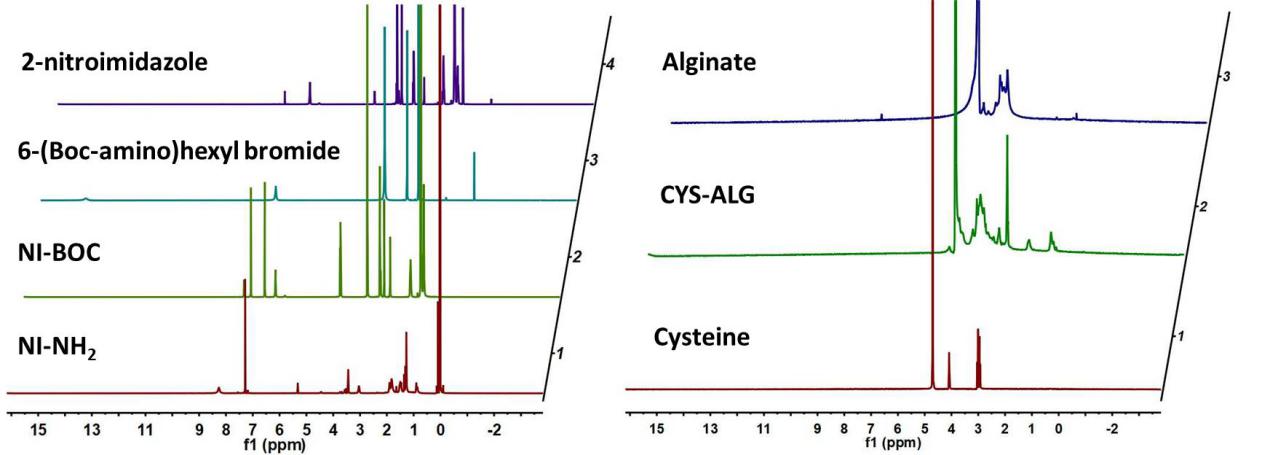


Figure S2 ^1^H NMR spectrum of 2-nitroimidazole, 6-(boc-amino)hexyl bromide, NI derivative (NI-BOC, NI-NH_2_), CYS, ALG and CYS-ALG.


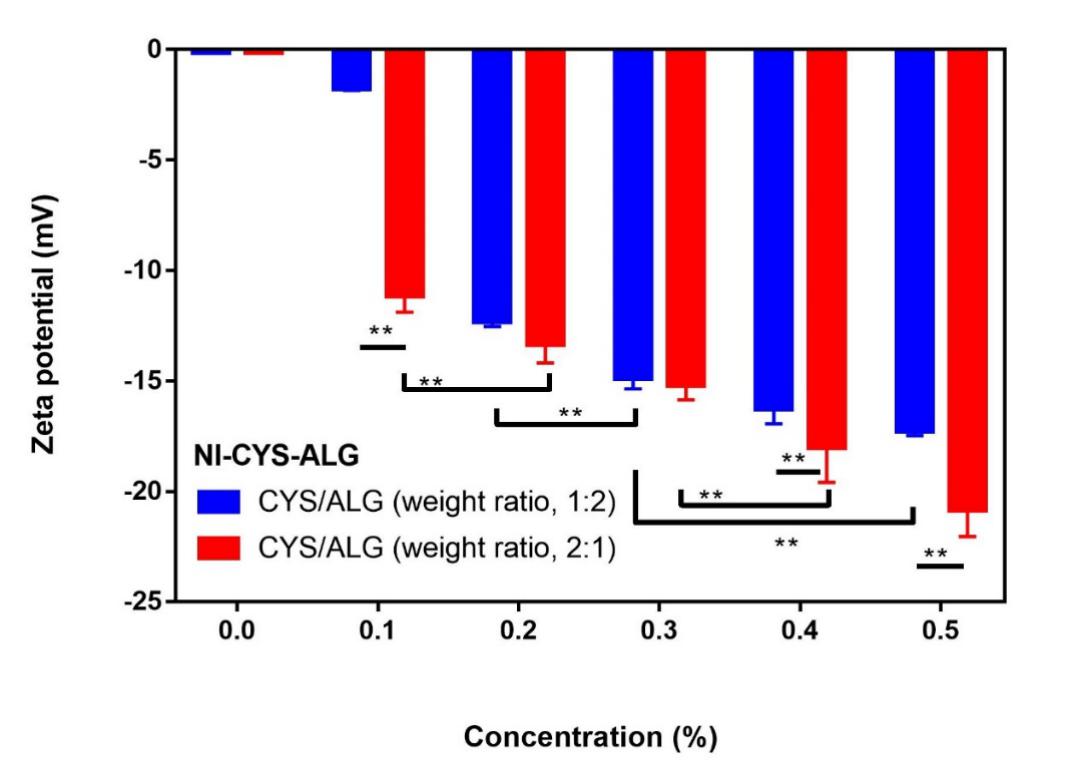


Figure S3 Zeta potential changes of NI-CYS-ALG conjugate under mucin particle adhesion method with different contents of cysteine (1:2-2:1, weight ratios).


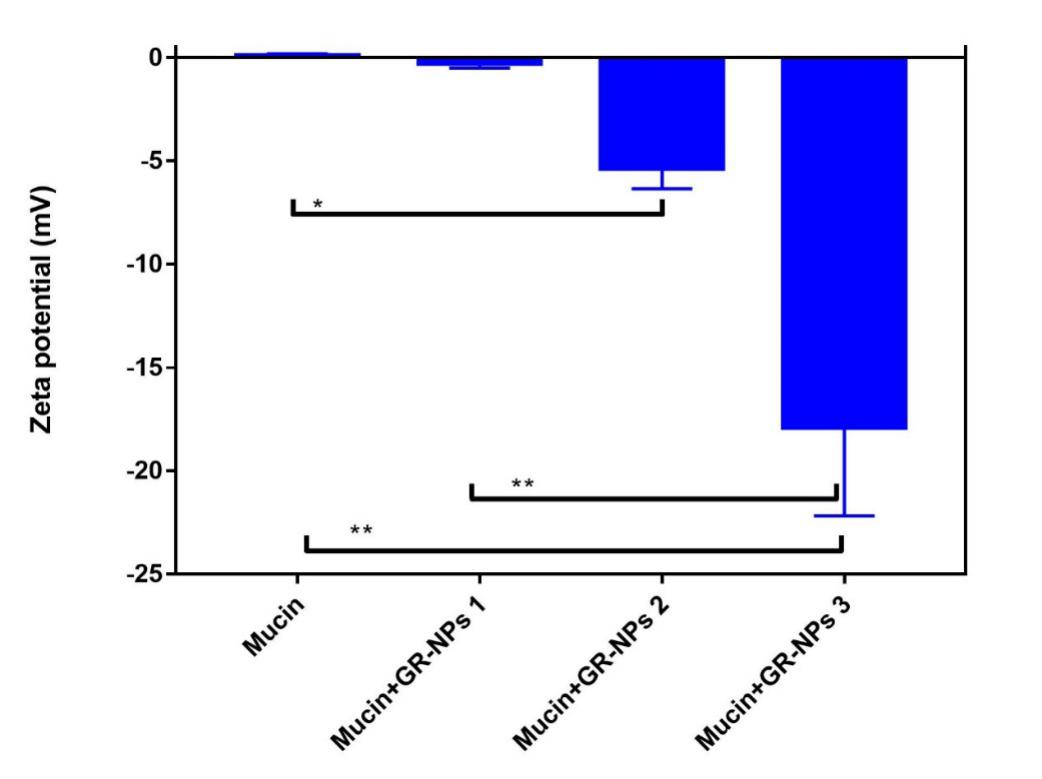


Figure S4 Zeta potential changes of GR-NPs under mucin particle adhesion method with different contents of cysteine (1:2, 1:1, 2:1, weight ratios).


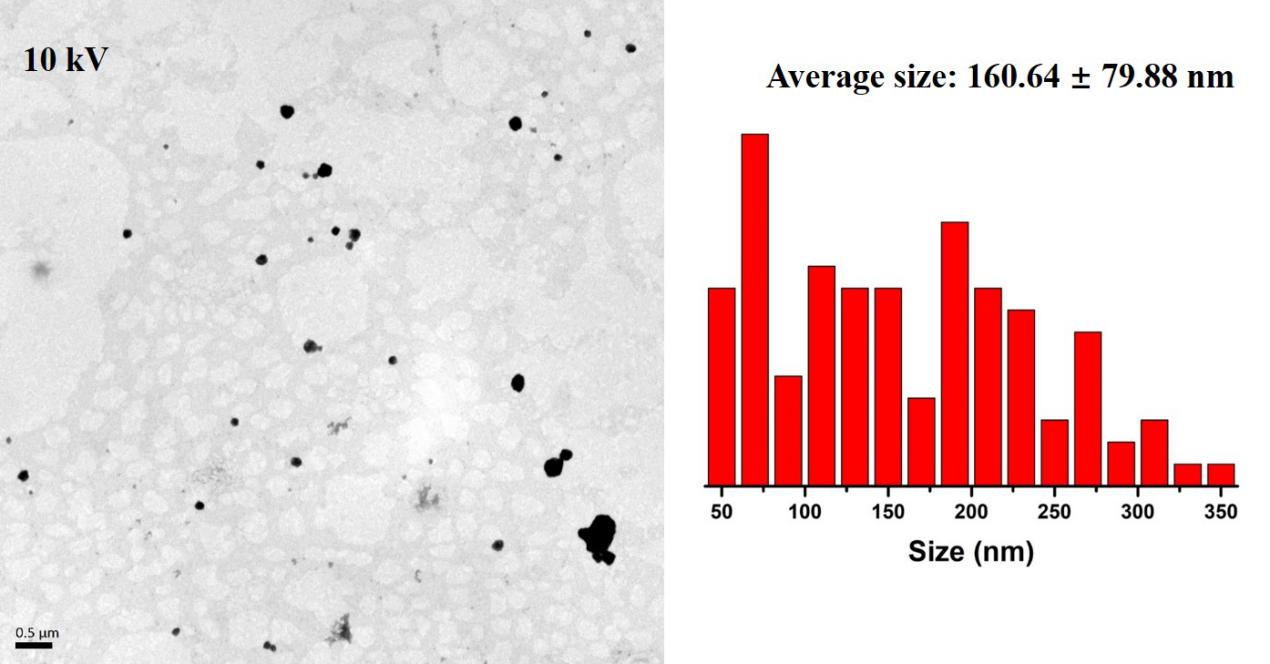


Figure S5 TEM images and size distribution of GR-NPs


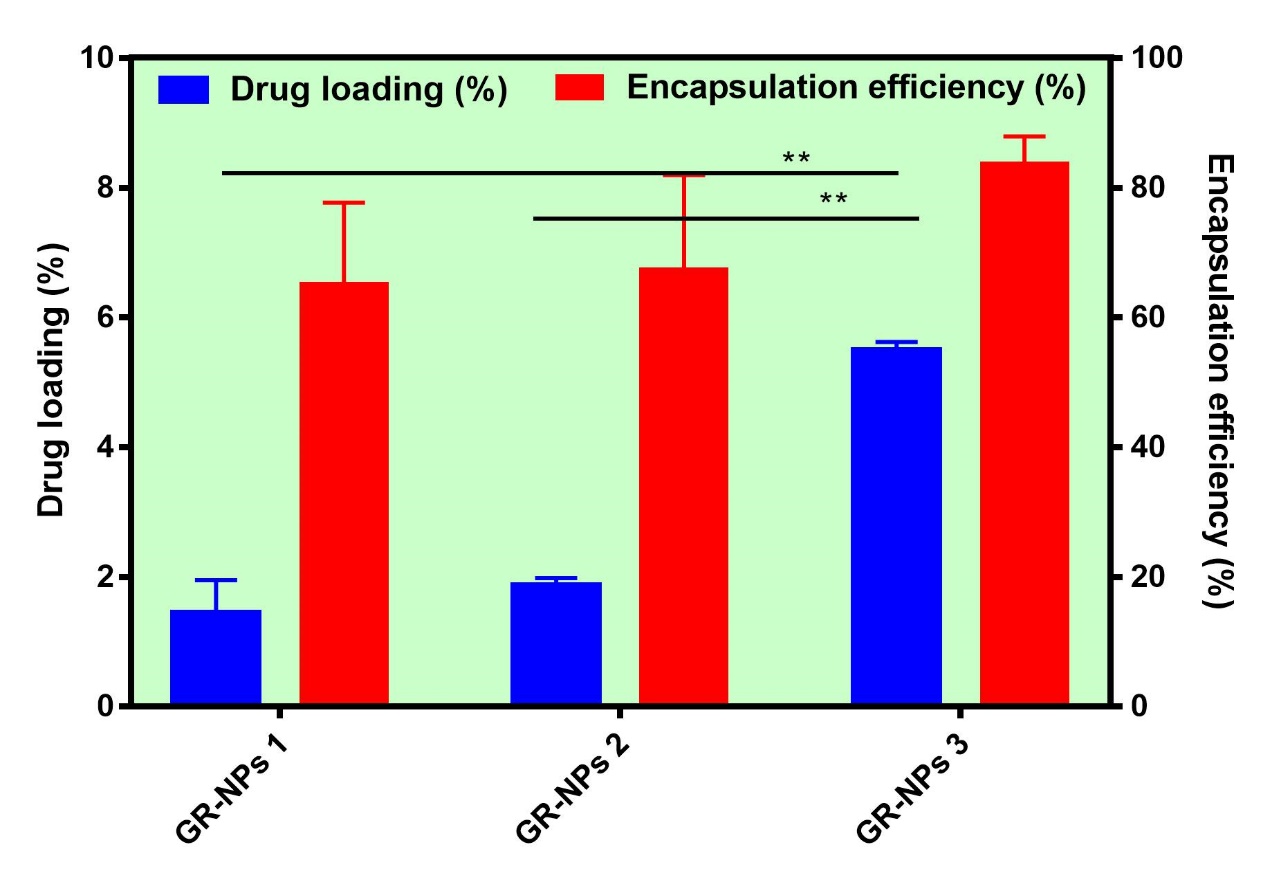


Figure S6 Drug loading and encapsulation efficiency of GR-NPs with different content of cysteine (1:2, 1:1, 2:1, weight ratios).


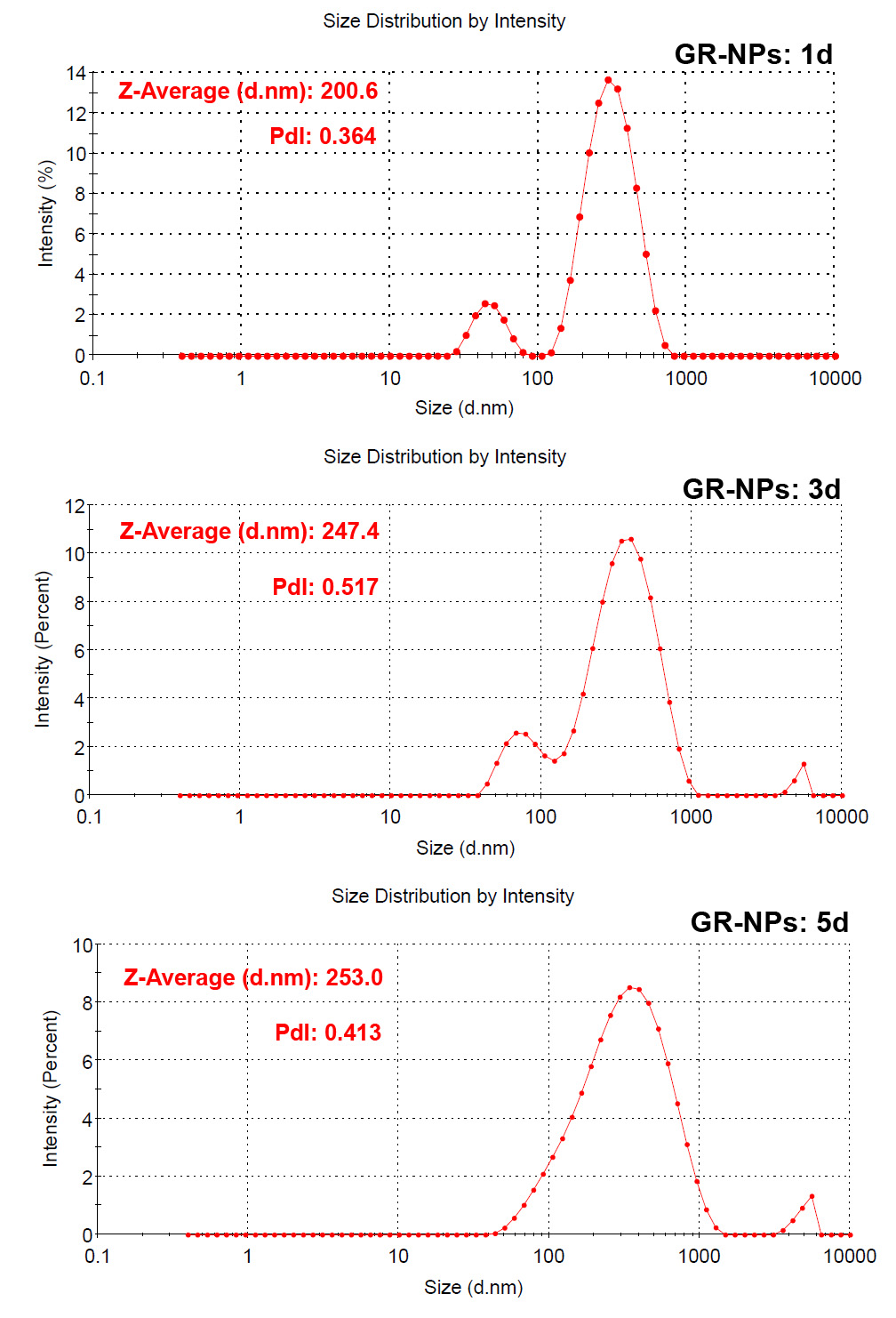


Figure S7 Particle size of GR-NPs in different days (1d, 3d, 5d).


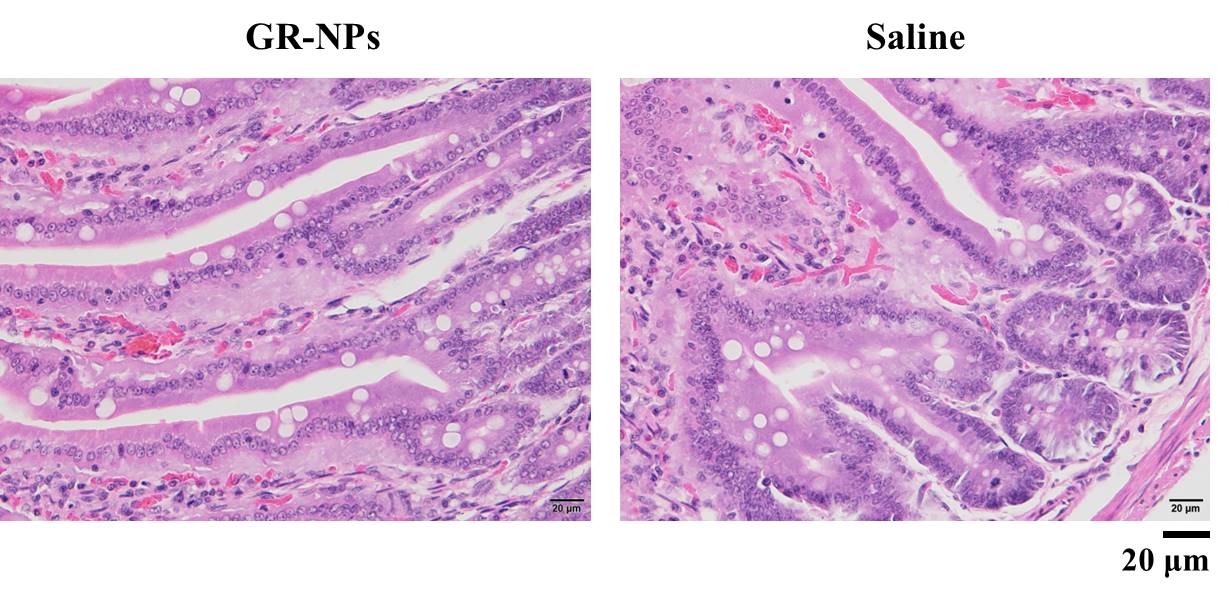


Figure S8 H&E staining observation of SD rat intestine after oral administration of GR-NPs and saline for 3 h.


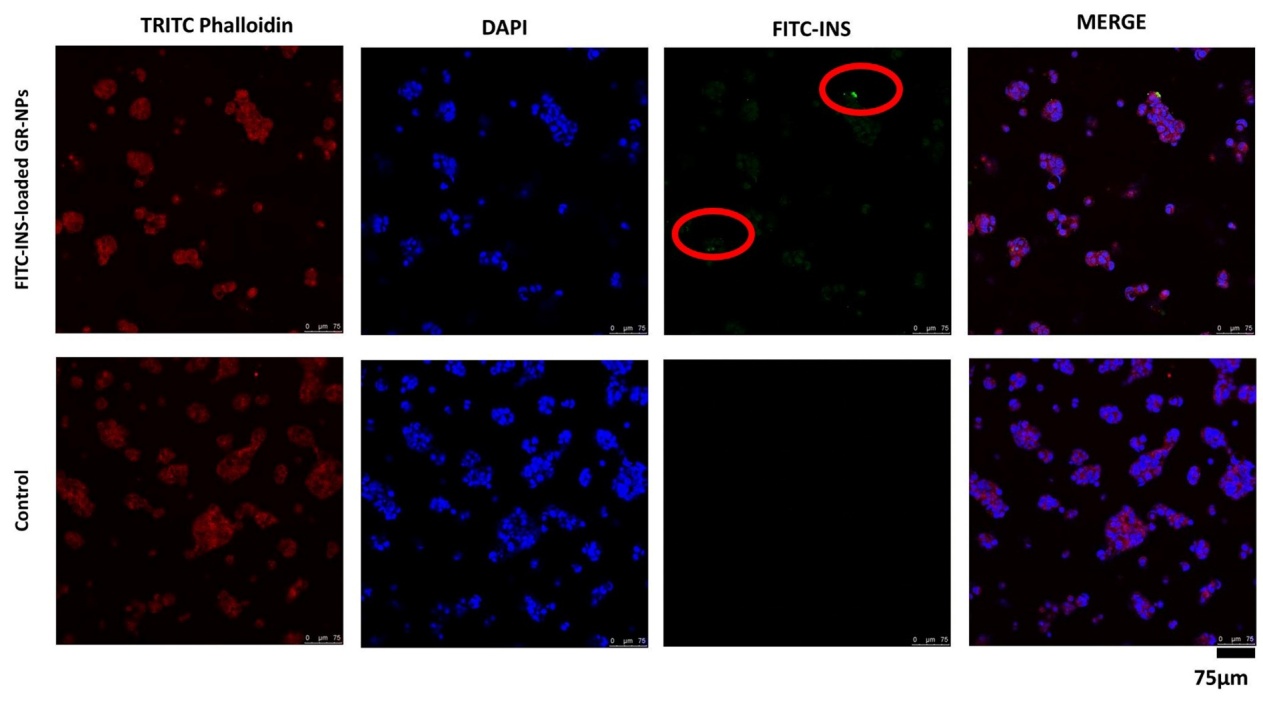


Figure S9 CLSM observation of cell adhesion with FITC-INS (control) and FITC-INS/GOx loaded GR-NPs after 30 min incubation.


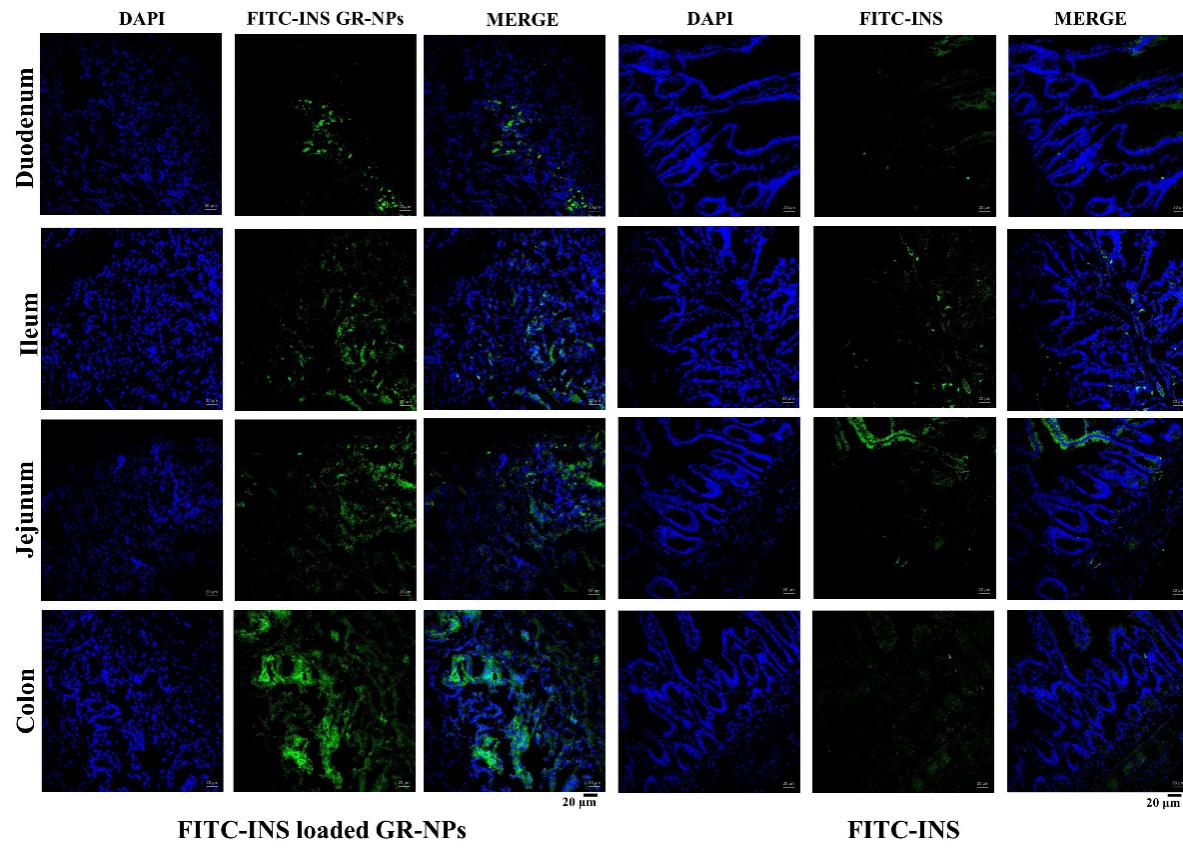


Figure S10 CLSM images of FITC-INS/GOx GR-NPs and FITC-INS in intestinal tissue (duodenum, ileum, jejunum and colon) of rats after 2 h oral administration.


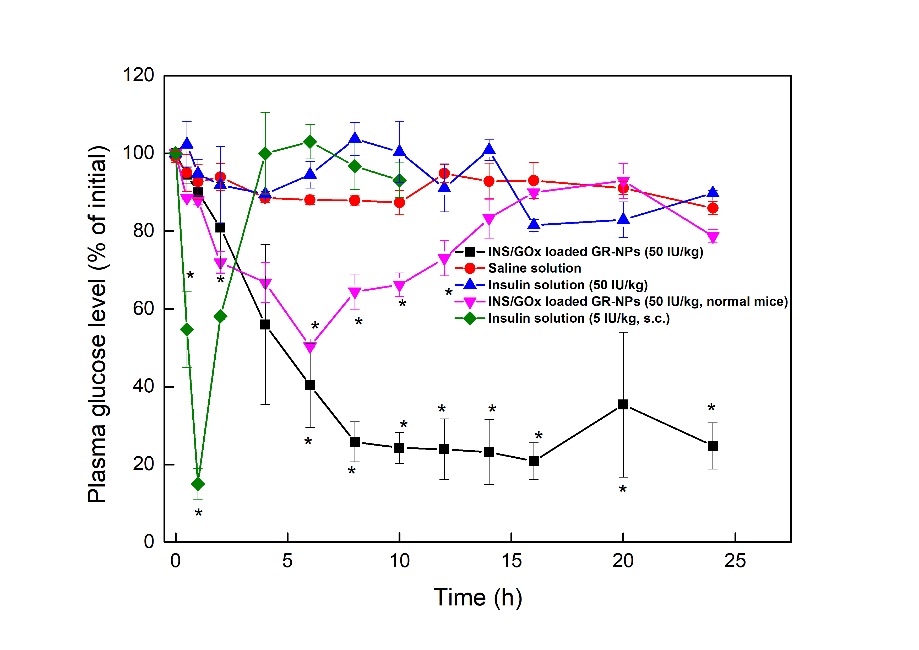


Figure S11 Plasma glucose levels in diabetic mice following oral administration of INS-loaded NPs, INS/GOx GR-NPs, saline or insulin solution, and following subcutaneous injection of insulin solution, and in normal rats following oral administration of INS/GOx GR-NPs.

Table S1 Concentration of CYS and NI in NI-CYS-ALG polymer with different weight ratios of CYS/ALG.

| Groups | CYS/ALG | | |
| --- | --- | --- | --- |
|  | 1:2 | 1:1 | 2:1 |
| CYS (μmol/g) | 227.77±5.31 | 241.63±7.86 | 332.06±6.31 |
| NI (μmol/g) | 272.93±4.81 | 240.41±10.02 | 310.39±6.59 |

Table S2 Particle size and Zeta potential of GR-NPs.

| Groups | CYS: ALG (weight ratio) | | |
| --- | --- | --- | --- |
|  | 1:2 | 1:1 | 2:1 |
| Size (nm) | 192.8 | 197.3 | 197.4 |
| PDI | 0.188 | 0.149 | 0.156 |
| Zeta (mV) | -40.8 | -41.5 | -41.6 |

**References**

1. Evans SM, Kim K, Moore CE, Uddin MI, Capozzi ME, Craft JR, et al. Molecular Probes for Imaging of Hypoxia in the Retina. Bioconjugate Chem. 2014;25(11):2030-37.

2. Li L, Jiang GH, Yu WJ, Liu DP, Chen H, Liu YK, et al. Preparation of chitosan-based multifunctional nanocarriers overcoming multiple barriers for oral delivery of insulin. Mat Sci Eng C-Mater. 2017;70:278-86.

3. Greimel A, Werle M, Kop-Schnurch AB. Oral peptide delivery: in-vitro evaluation of thiolated alginate/poly(acrylic acid) microparticies. J Pharm Pharmacol. 2007;59(9):1191-98.

4. He CB, Yin LC, Tang C, Yin CH. Size-dependent absorption mechanism of polymeric nanoparticles for oral delivery of protein drugs. Biomaterials. 2012;33(33):8569-78.

5. Madsen F, Peppas NA. Complexation graft copolymer networks: swelling properties, calcium binding and proteolytic enzyme inhibition. Biomaterials. 1999;20(18):1701-08.

6. Bravo-Osuna I, Millotti G, Vauthier C, Ponchel G. In vitro evaluation of calcium binding capacity of chitosan and thiolated chitosan poly(isobutyl cyanoacrylate) core-shell nanoparticles. Int J Pharm. 2007;338(1-2):284-90.

7. Matsushita K, Kinoshita K, Matsuoka T, Fujita A, Fujikado T, Tano Y, et al. Intramolecular interaction of SUR2 subtypes for intracellular ADP-induced differential control of K-ATP channels. CircRes. 2002;90(5):554-61.
